# Analysis of a novel mutant allele of *GSL8* reveals its key roles in cytokinesis and symplastic trafficking in Arabidopsis

**DOI:** 10.1186/s12870-018-1515-y

**Published:** 2018-11-22

**Authors:** Behnaz Saatian, Ryan S. Austin, Gang Tian, Chen Chen, Vi Nguyen, Susanne E. Kohalmi, Danny Geelen, Yuhai Cui

**Affiliations:** 1 Agriculture and Agri-Food Canada, London Research and Development Centre, London, ON Canada; 2 Department of Biology, Western University, 1391 Sandford St, London, ON N5V 4T3 Canada; 3 In Vitro Biology and Horticulture, Department of Plant Production, University of Ghent, 9000 Ghent, Belgium

**Keywords:** *Arabidopsis thaliana*, Callose, Cytokinesis, Callose synthase complex, GLUCAN SYNTHASE-LIKE 8, Intercellular signaling, Plasmodesmata, Symplastic trafficking

## Abstract

**Background:**

Plant cell walls are mainly composed of polysaccharides such as cellulose and callose. Callose exists at a very low level in the cell wall; however, it plays critical roles at different stages of plant development as well as in defence against unfavorable conditions. Callose is accumulated at the cell plate, at plasmodesmata and in male and female gametophytes. Despite the important roles of callose in plants, the mechanisms of its synthesis and regulatory properties are not well understood.

**Results:**

*CALLOSE SYNTHASE* (*CALS*) genes, also known as *GLUCAN SYNTHASE-LIKE* (*GSL*), comprise a family of 12 members in *Arabidopsis thaliana*. Here, we describe a new allele of *GSL8* (named *essp8*) that exhibits pleiotropic seedling defects. Reduction of callose deposition at the cell plates and plasmodesmata in *essp8* leads to ectopic endomitosis and an increase in the size exclusion limit of plasmodesmata during early seedling development. Movement of two non-cell-autonomous factors, SHORT ROOT and microRNA165/6, both required for root radial patterning during embryonic root development, are dysregulated in the primary root of *essp8*. This observation provides evidence for a molecular mechanism explaining the *gsl8* root phenotype. We demonstrated that GSL8 interacts with PLASMODESMATA-LOCALIZED PROTEIN 5, a β-1,3-glucanase, and GSL10. We propose that they all might be part of a putative callose synthase complex, allowing a concerted regulation of callose deposition at plasmodesmata.

**Conclusion:**

Analysis of a novel mutant allele of *GSL8* reveals that GSL8 is a key player in early seedling development in Arabidopsis. GSL8 is required for maintaining the basic ploidy level and regulating the symplastic trafficking. Callose deposition at plasmodesmata is highly regulated and occurs through interaction of different components, likely to be incorporated into a callose biosynthesis complex. We are providing new evidence supporting an earlier hypothesis that GSL8 might have regulatory roles apart from its enzymatic function in plasmodesmata regulation.

**Electronic supplementary material:**

The online version of this article (10.1186/s12870-018-1515-y) contains supplementary material, which is available to authorized users.

## Background

Plant cell walls are rich in polysaccharides such as cellulose and callose [1]. Even though in plants callose is accumulated in the cell wall at a lower ratio compared to cellulose, it plays significant roles [2]. Callose is required for cell plate formation during cytokinesis and its deposition and degradation at plasmodesmata (PD) are critical for regulation of symplastic trafficking [3–8].

Different from the other aspects of the cell cycle, cytokinesis is less conserved between non-plant organisms and higher plants. During cytokinesis in plants, at the end of anaphase, a tubulovesicular network is formed at the equator of dividing cells [9, 10]. Callose deposition at the tubulovesicular network enforces widening and consolidation of tubules which will consequently lead to conversion of the network into a fenestrated sheet [11]. Ectopic endopolyploidy caused by cytokinesis defects has been observed in different organisms and cell types [12–16].

Plant cells are immobile and therefore, positional cues and information exchange between cells are critical during plant development. Intercellular signaling processes occur through either apoplastic signaling, or symplastic movement of molecules via PD [17]. It has been proposed that callose deposition/degradation at the apoplastic neck of PD regulates its size exclusion limit (SEL) and, consequently, cell to cell connectivity [18, 19]. Although the important role of callose equilibration at PD in regulation of SEL and symplastic movement has been implicated [20], the molecular mechanism(s) linking the identified players for endogenous signaling to callose homeostasis is largely unknown.

The Arabidopsis genome has 12 genes encoding GLUCAN SYNTHASE-LIKE (GSL) [21], also called CALLOSE SYNTHASE (CALS) [22]. GSL enzymes synthesize callose in response to different developmental, physiological, and environmental signals and in various plant tissues [22–27]. Out of the 12 GSLs in Arabidopsis, GSL4, GSL6, GSL7, GSL8 and GSL12 have so far been indicated to be associated with plasmodesmata regulation [28–31].

*GLUCAN SYNTHASE-LIKE 8* (*GSL8*) is one of the few members of the *GSL* family with high expression during plant development [32]. *gsl8* mutants exhibit pleiotropic defects and lethality [30, 33–36], but the mechanisms underlying these phenotypes remain mostly unknown.

Here, we report a new mutant allele of *GSL8* called *essp8*, identified in a genetic screen for mutations inducing the ectopic expression of the seed storage proteins (*essp*) [37–42]. We provide new experimental evidence suggesting that *gsl8*/*essp8* developmental defects are caused by both cytokinesis impairments and dysregulation of symplastic trafficking via PD.

## Results

### Developmental defects in *essp8* seedlings are caused by a splice site mutation in *GSL8*

**Fig. 1:**
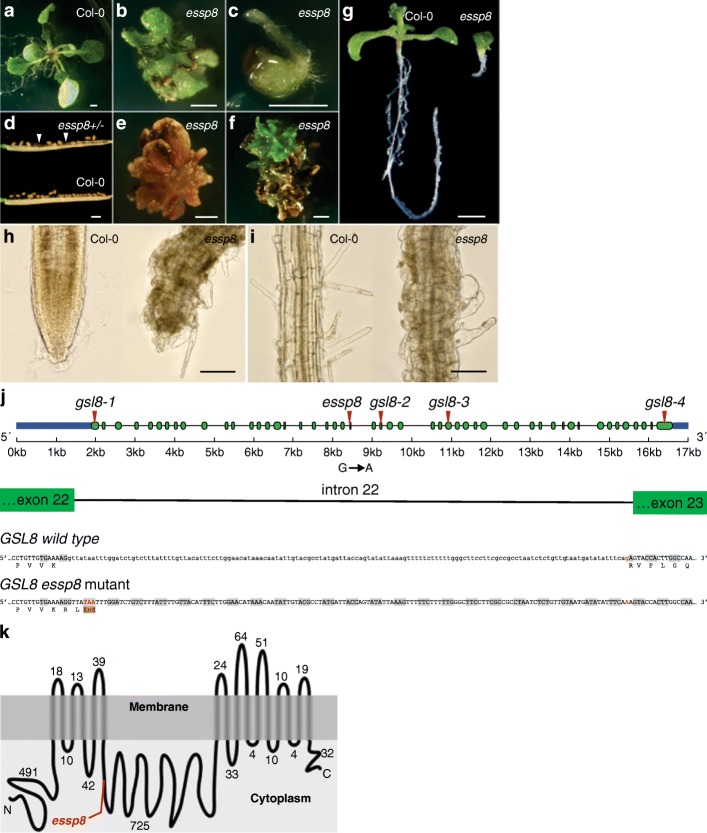
Morphological phenotype of the *essp8* mutant. **a**-**c** Comparison of seedling phenotypes of WT Col-0 (**a**) and *essp8* mutants (**b**-**c**) grown on MS agar for two weeks. The *essp8* roots and hypocotyls are shorter and thicker compared to the WT Col-0. **d** Siliques from a heterozygous parent showing the formation of defective seeds (white arrowheads). **e** Representative image showing the seedling lethal phenotype in a 3-week-old *essp8* mutant. **f** Image showing the ectopic cell proliferation in a small percentage of mutants (10%). **g** Ten-day-old WT and *essp8* mutant seedlings showing the stunted roots in the mutant. **h**-**i** Comparison of the root phenotype between five-day-old WT and *essp8* mutant seedlings at the root tip (**h**) and elongation zone (**i**) showing abnormally-developed root tips, having bloated cells and formation of short, swollen, and often branched root hairs in the elongation zone. **j** *GSL8* gene structure showing exons (green boxes) and introns (lines). The mutation/insertion sites (red triangles) of alleles used in this work are indicated. The splice site at intron 22 is disrupted in *essp8* causing a retention of intron 22 in *essp8* which establishes a premature STOP-codon downstream of exon 22. **k** *GSL8* encodes a large transmembrane protein. GSL8 has a large cytoplasmic central loop between the transmembrane domains. The *essp8* mutation results in the truncation of the GSL8 protein at the fifth cytoplasmic helix, identified by the red line. Scale bars = 1 mm (**a**-**f**), 200 μm (**g**), 50 μm (**h**-**i**)

*essp8* seedlings exhibit several developmental defects including dwarfism, formation of abnormally-developed cotyledons and true leaves, reduced growth of the root and hypocotyl, and generally delayed development compared to wild type (WT) Col-0 (Fig. 1a-c). The *essp8* mutation causes incomplete embryo lethality (~ 20% of the homozygous seeds failed to germinate) and thus reduced transmission in the progeny (See Additional file 1: Table S1). Examination of the siliques from a heterozygous parent show that ~ 25% of the seeds are visually defective, being smaller, darker and shrunk compared to wild-type seeds (Fig. 1d; See Additional file 1: Table S2). The *essp8* mutation is lethal in most of the mutant seedlings, leading to their death after three weeks (Fig. 1e). However, it can induce ectopic cell proliferation in the seedlings that survive longer (Fig. 1f). *essp8* mutants show severe defects in root tissue patterning (Fig. 1g) with bloated cells, loss of radial patterning, and develop short, swollen and often branched root hairs (Fig. 1h-i).

The *essp8* mutation was mapped on the bottom arm of chromosome 2 (See Additional file 2**:** Figure S1A-C) and a single non-synonymous EMS-induced point mutation (G/C to A/T substitution) was identified in AT2G36850 (*GSL8*) (See Additional file 2: Figure S1D). The mutation disrupts the splice site of *GSL8* at intron 22, introducing a premature STOP-codon (Fig. 1j). Disruption of *GSL8* mRNA downstream of exon 22 was confirmed using RT-PCR (data not shown). GSL8 is a large integral membrane protein predicted to form sixteen transmembrane helices [43]. The transmembrane domains are clustered into an N-terminal and a C-terminal region leaving a large hydrophilic central loop within the cytoplasm (Fig. 1k). In *essp8*, introduction of the premature STOP-codon results in the truncation of the GSL8 protein at the fifth cytoplasmic hydrophilic loop (Fig. 1k).

To confirm that *ESSP8* is indeed allelic to *GSL8*, four T-DNA insertion lines, SALK_11500 (*gsl8–1*), SALK_109342 (*gsl8–2*), SAIL_21_B02 (*gsl8–3*) and SALK_098374 (*gsl8–4*), were obtained (Fig. 1j). Homozygous T-DNA mutant seedlings for all four lines exhibited similar phenotypes as that of *essp8* (See Additional file 2: Figure S2A-F and S3). Similar to *essp8*, the T-DNA alleles also showed reduced transmission in the progeny (See Additional file 1: Table S3). An allelism test was performed. F1 progeny seedlings heterozygous for two different mutant alleles of *GSL8* recapitulated the morphological phenotype of homozygous *gsl8* or *essp8* mutants (See Additional file 2: Figure S2G-I). Genetic transformation of the *essp8* mutant with the GSL8 coding sequence driven by its native promoter successfully rescued the mutant phenotype (See Additional file 2: Figure S4). These observations demonstrate that *essp8* is indeed a new allele of *GSL8*.

### Callose deposition at both cell plate and plasmodesmata is decreased in *essp8* roots

**Fig. 2:**
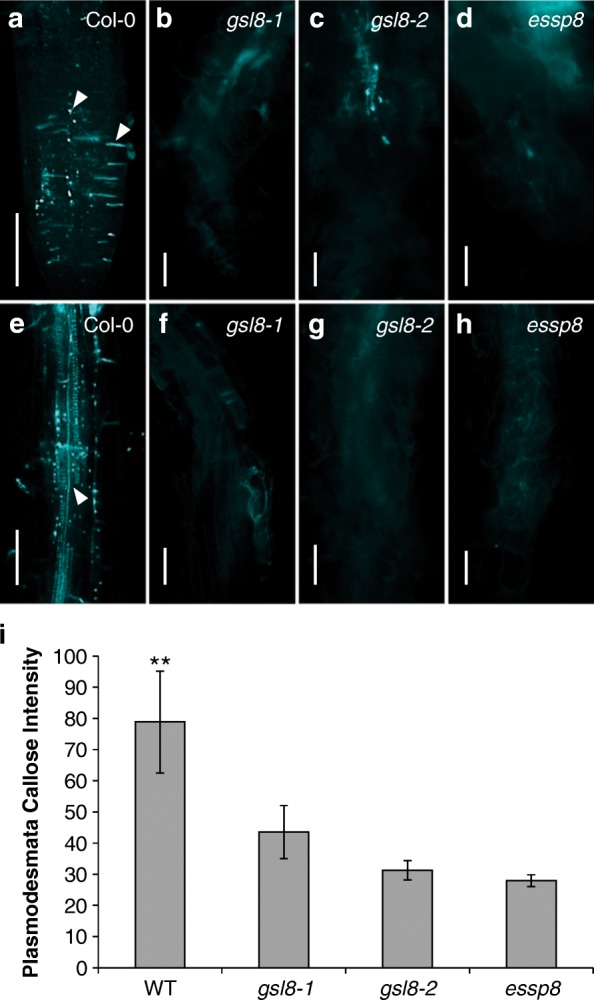
Callose deposition in the primary root of *gsl8* mutants. Callose accumulation was visualized using aniline blue staining in five-day-old seedlings of WT, *gsl8–1*, *gsl8–2* and *essp8*. **a**-**d** Bright blue lines and dots (white arrowheads) at the root tip represent callose deposition at the cell plate and plasmodesmata, respectively, in WT (**a**) and *gsl8* mutants (**b**-**d**). **e**-**h** In the elongation zone of the primary root, callose deposition at plasmodesmata is detected as blue dots in the root hairs, cell wall and vascular tissue (white arrowhead) in the WT (**e**) and *gsl8* mutants (**f**-**h**). **i** Quantification of callose levels in *gsl8* mutants compared to WT Col-0. Values represent mean ± SEM (*n* = 10). **P < 0.01. Scale bars = 100 μm

During plant growth and development, callose is accumulated in different tissues and cells, where it plays vital roles. Callose deposition at the cell plate and PD is required for completion of cytokinesis and physical constriction of PD, respectively [30, 35, 36]. The effect of the *gsl8* mutation on callose deposition at the cell plate and PD was investigated using the callose-specific dye, aniline blue fluorochrome, in the primary root of WT Col-0, *essp8*, *gsl8–1* and *gsl8–2*. In the WT root tip, bright, linear signals, representing the callose deposited at the cell plate [30] in newly divided cells, were detected (Fig. 2a). Concomitantly, the punctate fluorescent signals at the cell walls, root hairs and vascular tissues in the elongation zone of the root (Fig. 2a and e) indicate callose deposition at PD [44]. In contrast to the WT, *essp8*, *gsl8–1* and *gsl8–2* roots showed weaker signal at the cell plates (Fig. 2b-d), PD (Fig. 2f-h) and in the root hairs and vasculature tissue. Quantification of callose accumulation at PD demonstrated significant reduction of callose signal in all three *gsl8* mutants compared to the WT (Fig. 2i). We thus conclude that GSL8 plays important role in callose biosynthesis and deposition at cell plates and PD in the primary root of Arabidopsis seedlings.

### GSL8 is required for the completion of cytokinesis in embryonic root

Formation of defective cell plate during cytokinesis can cause abortion of cell division. Depending on the stage when mitosis is aborted, separation of the newly-formed nuclei or duplicated chromosomes is disrupted, leading to generation of multi-nucleated cells or cells with doubled chromosome numbers known as endomitosis [45]. To investigate whether the severe phenotypic defects in *essp8* seedlings are caused by incomplete cytokinesis, different known cytokinesis-defective mutants: *hinkel* [46], *knolle* [47], *keule* [48], *korrigan* [49] and *stomatal cytokinesis-defective 1* (*scd1*) [50], were examined for their morphological phenotypes. All the tested cytokinesis-defective mutants are dwarf, reminiscent of *essp8* seedling morphology (See Additional file 2: Figure S5A-F). *knolle*, *keule* and *korrigan* form fused cotyledons and fail to develop true leaves (See Additional file 2: Figure S5D-F). *hinkel* and *scd1*, similar to *essp8* seedlings*,* form short roots and thicker leaves (See Additional file 2: Figure S5B-C).

**Fig. 3:**
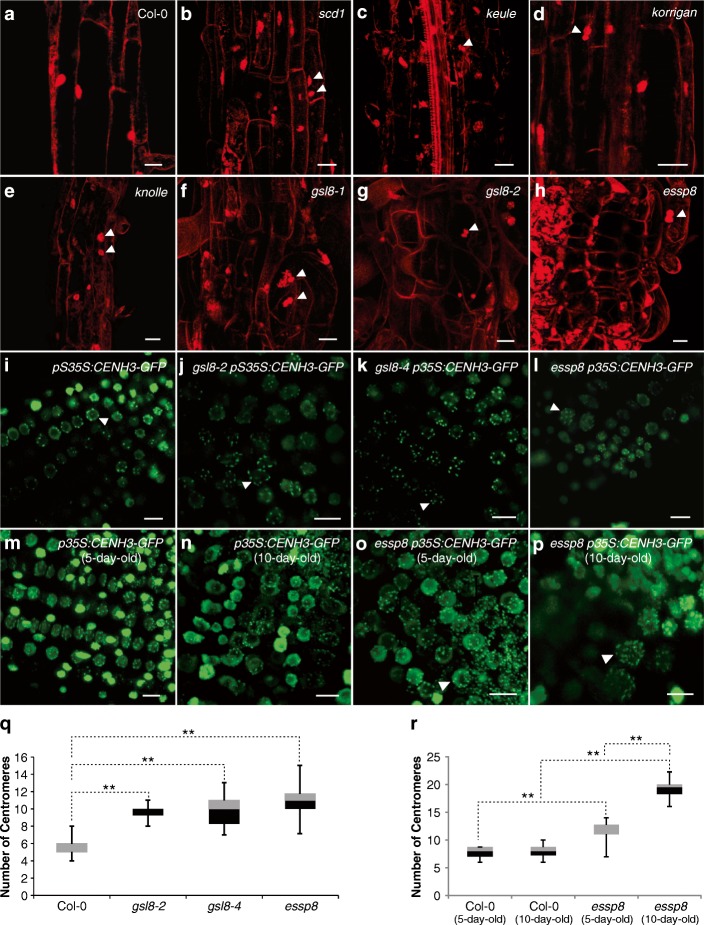
Formation of binucleated and endomitotic cells in the primary root of *gsl8* mutants. **a**-**h** Comparison of the number of nuclei per cell in the elongation zone of the root between WT (**a**), cytokinesis-defective mutants; *scd1* (**b**), *keule* (**c**), *korrigan* (**d**) and *knolle* (**e**), and *gsl8* mutants; *gsl8–1* (**f**), *gsl8–2* (**g**), *essp8* (**h**). Binucleated cells are shown by white arrowheads. Propidium iodide was used to stain both nuclei and cell walls. **i**-**l** Comparison of centromere numbers (white arrowheads) in the primary roots of WT (**i**) and *gsl8* mutants; *essp8* (**j**), *gsl8–2* (**k**) and *gsl8–4* (**l**). **m**-**p** Comparison of polyploid cells in *essp8* mutant at different ages in the primary root cells of five- and ten-day-old WT (**m-n**), and *essp8* (**o**-**p**), respectively. Polyploid cells are shown by white arrowheads. **q** Quantification of the centromere number per nucleus in the WT and three *gsl8* mutants. **r** Quantification of centromere numbers in five- and ten-day-old primary root of WT and *essp8*. **q**-**r** Data were acquired from at least ten cells in an individual seedling and three biological replicates. The boxes signify the upper (grey) and lower (black) quartiles, and the median is represented by a short black line within the box for each plant line. The upper and lower “whiskers” represent the entire spread of the data. Dotted lines indicate significant differences (**P < 0.01 using Student’s t-Test) between the two samples. Scale bars = 20 μm (**a**-**h**), 10 μm (**i-p**)

Bi- or multi-nucleated cells are formed where two daughter cells fail to separate by a cross-wall. To explore if *essp8* mutant seedlings form binucleated cells, the primary root was stained with propidium iodide (PI). Binucleated cells were observed in *scd1*, *keule*, *korrigan* and *knolle* (Fig. 3a-e). Similar to the cytokinesis-defective mutants, *gsl8* seedlings also have cells with more than one nucleus suggesting that *gsl8* can be categorized as a cytokinesis-defective mutant (Fig. 3f-h). However, a very frequently observed defect in *essp8* root (enlarged disorganized cells with abnormal shapes) was not observed in cytokinesis-defective mutants. This result indicates that *essp8* morphological and developmental defects are only partially attributable to cytokinesis impairments.

Identification of binucleated cells in somatic tissues of *essp8* root prompted us to investigate the possibility of endomitosis. Using a centromere-labeling construct, *p35S:CENH3-GFP* [35, 51], the absolute number of chromosomes was counted in *gsl8* mutant backgrounds in vivo. The diploid status of the epidermal cells in WT primary root was confirmed by detection of 5 to 10 centromeric dots (Fig. 3i). Different from WT, *essp8*, *gsl8–2* and *gsl8–4* nuclei with higher number of chromosomes (ranging from 11 to 15) were observed, indicating the presence of triploid and potentially polyploid cells in these mutants (Fig. 3j-l). Comparing the centromere numbers between WT and *gsl8* mutants revealed a significant increase in all three *gsl8* mutants studied (Fig. 3q). Therefore, we conclude that the *essp8* mutation can induce not only ectopic endomitosis in reproductive cells (previously shown by De Storme et al. 2013), but also in the somatic root cells at very early stages of development.

Furthermore, we examined whether polyploidization level increases with age in the *essp8* mutant. Five-day-old *essp8* roots harbored scattered single enlarged endomitotic and/or polyploid cells. Wild-type root cells were homogeneously sized containing single diploid nuclei (Fig. 3m-n and r). In contrast, a marked elevation in the number of chromosomes within the cells was observed in ten-day-old *essp8* seedlings (Fig. 3o, p and r). This result suggests that with age, seedling phenotype deterioration accompanies a significant increase in polyploidization defects which may lead to seedling lethality later on.

### GSL8 regulates symplastic connectivity through plasmodesmata

Callose deposition is a highly-regulated dynamic process, which is required for adjustment of PD SEL in response to endogenous and exogenous signals [20]. Earlier, the aniline blue staining indicated the requirement of GSL8 for callose deposition at PD (Fig. 2). Thus, it was postulated that decrease in callose accumulation at PD in *essp8* leads to an increase in PD SEL. To test this hypothesis, passive cell-to-cell diffusion of two fluorescent probes, Alexa flour and fluorescein (3 kDa and 10 kDa in size, respectively), was investigated in *essp8* hypocotyls, as previously described [52]. The fluorescent probes were separately injected into the hypocotyls (See Additional file 2: Figure S6A-B). Diffusion of fluorescent signal was measured as the distance between the injection site and the furthest detected signal right after injection. For the smaller probe (Alexa Fluor), fluorescent signal was detected at the site of injection and surrounding cells in both WT and *essp8* (See Additional file 2: Figure S6C-D). However, the distance of its movement was significantly longer in *essp8* (See Additional file 2: Figure S6G). In contrast, the larger probe (fluorescein) was only detected at the site of injection in nearly all cases in WT. Only a few surrounding cells showed a dim fluorescent signal, indicating its limited diffusion in WT hypocotyls (See Additional file 2: Figure S6E). The injected *essp8* hypocotyls showed strong fluorescent signal in many more of the surrounding cells (See Additional file 2: Figure S6F) and a longer traveling distance away from the injection site (See Additional file 2: Figure S6G). Diffusion pattern of Alexa Fluor in both WT and *essp8* suggests that its size (3 kDa) is below the PD SEL in hypocotyls, whereas Lack of fluorescein diffusion in the WT proposes that 10 kDa is possibly beyond the SEL. These results provided preliminary evidence that reduction of callose deposition at PD results in an increase in SEL in the *essp8* hypocotyl.

### SHORT ROOT and miR165/6 movements through plasmodesmata are dysregulated in *essp8*

During root development in Arabidopsis, the endodermis, middle cortex, and cortex are formed by timely and spatially regulated periclinal cell divisions. The formation of endodermis and cortex occurs continuously by parallel division of the cells surrounding the quiescent centre (QC) at the root tip, which is mediated by the activities of two transcription factors, SHORT ROOT (SHR) and SCARECROW (SCR) [53–55]. SHR has the ability to move from the stele cells, its domain of transcription, to a single layer of adjacent cells, and all endodermis cells [56, 57]. SHR movement acts both as a signal from the stele and an activator of endodermal cell identity determination and cell division through the transcriptional activation of *SCR* [56].

**Fig. 4:**
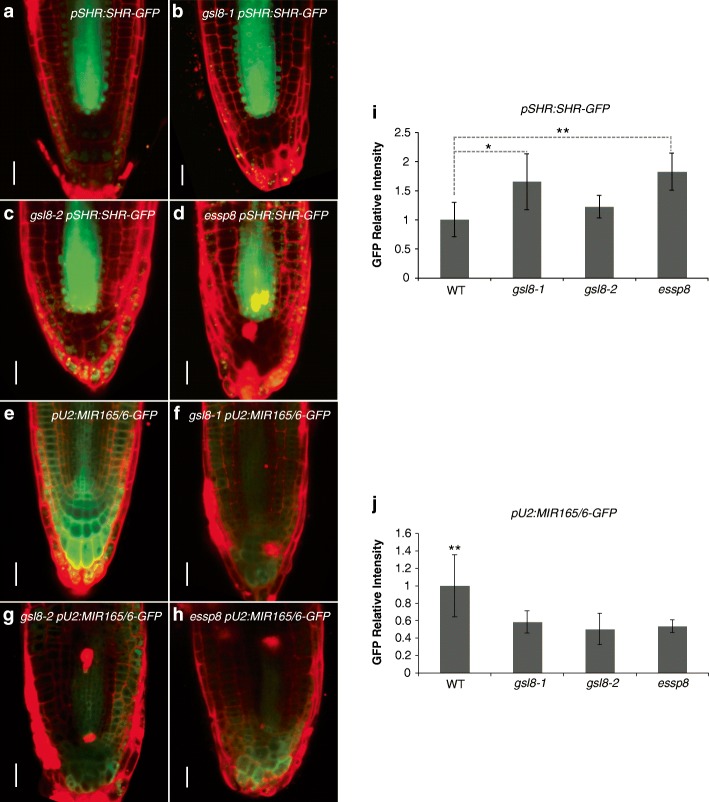
Symplastic movement of SHR and miR165/6 is altered in *essp8*. **a**-**d** Localization of SHR-GFP fusion proteins in the primary root of three-day-old WT (**a**), *gsl8–1* (**b**), *gsl8–2* (**c**) and *essp8* (**d**). **e**-**h** miR165/6 activity in transgenic lines with *pU2:MIR165/6-GFP* in primary root of three-day-old WT (**e**), *gsl8–1* (**f**), *gsl8–2* (**g**) and *essp8* (**h**). Roots were stained with PI to visualize the cell wall. Scale bars = 20 μm. **i**-**j** Measurement of GFP ratio in endodermal cells to stele relative to the WT (**i**) and quantification of GFP intensity in *gsl8* mutants’ primary root in *gsl8* mutants (**j**) relative to WT. Values represent mean ± SEM (n = 10), *P < 0.05, **P < 0.01

In *essp8*, embryonic root harbors disorganized cells and defective radial patterning (Fig. 1h-i). To explore whether the increased SEL of PD and root tissue patterning are related, SHR symplastic movement was investigated in *gsl8* mutants. A GFP-tagged version of SHR was expressed under its native promoter (*pSHR:SHR-GFP*) in WT, *gsl8–1*, *gsl8–2* and *essp8* plants. In three-day-old WT and *gsl8* mutant seedlings, SHR-GFP is localized into both the nucleus and cytoplasm of stele cells, but only the nucleus of neighboring cell layers including the QC, cortex/endodermis initial (CEI), and endodermis (Fig. 4a-d). The level of SHR-GFP signal was measured in the endodermis as a percentage of the stele signal in *gsl8* mutants relative to the WT. This method has been previously implicated as a good indication of SHR movement [58, 59]. In all *gsl8* mutants, the endodermal GFP signal to stele was higher than WT with significant increase found in *essp8* (Fig. 4i), indicating that SHR cell-to-cell movement is dysregulated in *essp8* root. Furthermore, the transcriptional activation of *SCR* in the endodermis requires SHR [60]. Since symplastic movement of SHR is affected in *essp8*, *SCR* expression is expected to be upregulated in *essp8* embryonic roots. Indeed, elevated level of *SCR* transcript was detected in both five-day- and ten-day-old *essp8* roots (See Additional file 2: Figure S7). We conclude that dysregulation of PD-mediated movement of SHR leads to the ectopic activity of SHR and upregulation of *SCR* during early seedling development in Arabidopsis.

miR165/6 species are transcribed in the endodermis outside of the stele, and then move from the endodermis into the stele where they target the transcripts of class III homeodomain leucine zipper (*HD*-*ZIP III*) family genes [61, 62]. To further explore whether the defective root tissue patterning in *essp8* is, at least partially, the result of dysregulated symplastic signaling, miR165/6 activity in the endodermis and stele of *gsl8* mutant primary roots was investigated using a ‘miRNA-sensor’ system as previously described [61, 63]. In this system, lower GFP expression is an indicative of higher miRNA activity. In WT, the GFP signal was weak in the stele and endodermis, confirming the expected miR165/6 activity in these cell layers (Fig. 4e). The GFP signal intensity was higher in the QC, lateral and columella root cap, indicating the absence of miR165/6 activity in these tissues (Fig. 4e). In all the three *gsl8* mutants, weak GFP signal was observed in the stele and endodermis (Fig. 4f-h). Notably, miR165/6 was also found to be ectopically active in the outer cell layers, as evidenced by the weak GFP signals in the epidermis, QC, and lateral and columella root cap (Fig. 4f-h). Measuring the GFP intensity in *gsl8* mutants’ roots relative to the WT indicates significant decreases in all the three mutants (Fig. 4j).

**Fig. 5:**
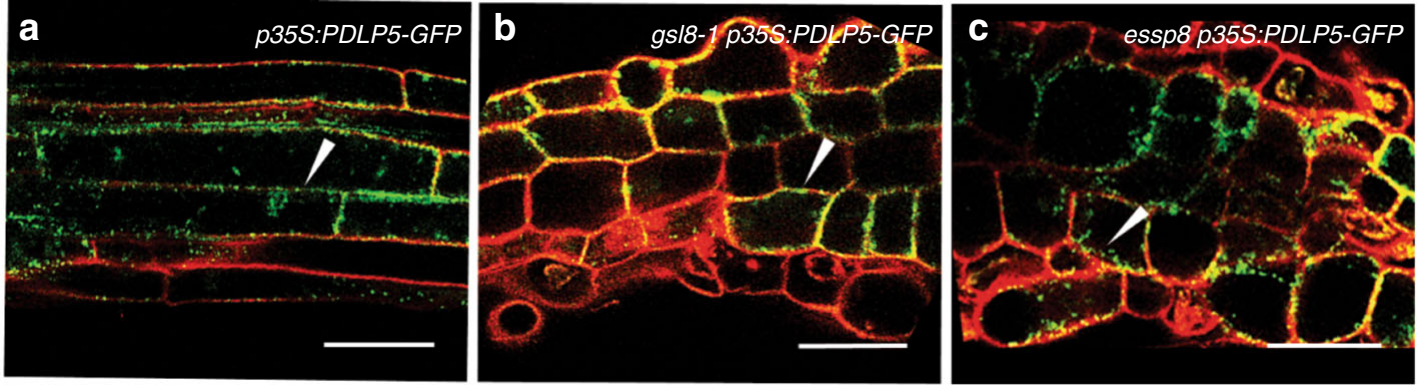
Subcellular localization of PDLP5-GFP in WT and *gsl8* primary root. **a**-**c** In the WT background, PDLP5-GFP is localized at the cell membranes in a dotted pattern in the primary root (**a**). Similarly, in the *gsl8–1* (**b**) and *essp8* (**c**) backgrounds, the PDLP5-GFP signal can be detected at the cell periphery (white arrowheads). Scale bars = 50 μm

To determine that defective cell plate formation in *essp8* seedlings is not causing the impairment of PD biogenesis, we used a PD marker, PLASMODSMATA-LOCALIZED PROTEIN 5 (PDLP5), to test its localization in the WT and *essp8*. The *p35S:PDLP5-GFP* construct was introduced into WT Col-0, and heterozygous *GSL8/gsl8–1* and *GSL8/essp8* plants. PDLP5-GFP signals were detected at punctate particles at the cell membrane in the elongation zone of their roots, suggesting that it is associated with the PD apertures [64] and PD biogenesis is not affected by defective cell plate formation in *gsl8* mutants [65] (Fig. 5a-c). Taken together, these results reveal that loss of callose deposition at PD in *essp8* seedlings causes relaxation of PD-mediated intercellular signaling.

### GSL8 interacts with plasmodesmata localized proteins

**Fig. 6:**
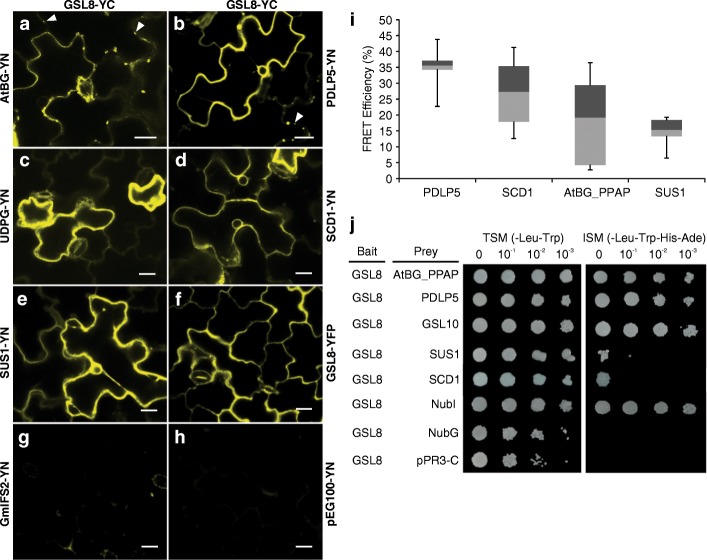
Analysis of GSL8 physical association with proteins involved in callose synthesis. **a**-**f** BiFC assay showing the interaction between YC fusion of GSL8 and YN fusions of AtBG_PPAP (**a**), PDLP5 (**b**), UDPG (**c**), SCD1 (**d**) and SUS1 (**e**). Interactions between GSL8 and AtBG_PPAP/ PDLP5 appear to be localized at the cell membrane and plasmodesmata (white arrowheads) (**a-b**). GSL8 interacts with UDPG and SUS1 in the cytoplasm and on the ER (**c** and **e**). Interaction of GSL8 with SCD1 occurs at the cell membrane and the cell plate (**d**). Subcellular localization of GSL8-YFP fusion shows its localization at the cell membrane (**f**). There was no interaction between GSL8-YC and GmIFS2-YN, an ER membrane-localized protein from soybean [70], and pEG100-YN which were used as negative control (**g**-**h**). Scale bars = 20 μm. **i** FRET confirms GSL8 interaction with PDLP5, SCD1, AtBG_PPAP and SUS1. No interaction was found with UDPG and free CFP (data not shown). The boxes signify the upper (dark grey) and lower (light grey) quartiles, and the median is represented by a short black line within the box for each. The upper and lower “whiskers” represent the entire spread of the data. The data used for FRET efficiency calculation and statistical analysis were obtained from three independent experiments and three biological replicates for each experiment. In all cases, values reported are the mean ± SEM. **j** MYTH assay. Colonies of transformed yeast cells growing on transformation selection media (TSM) and interaction selection media (ISM) indicate successful interaction of bait (GSL8) and prey (AtBG_PPAP, PDLP5, GSL10, SUS1 and SCD1). NubI was used as a positive control. NubG and empty vector were used as negative controls

It has been predicted that GSL might be part of a hypothetical complex called callose synthase complex (CalS) [34]. To investigated the composition of this complex, we selected a few candidates to test their possible interaction with GSL8 as follows: 1) previously suggested components of CalS, including at least one of the GSLs, UDP-glucose transferase 1 (UGT1) and sucrose synthase (SuSy) [24, 66–68]; 2) proteins proposed to be involved in PD SEL regulation, including callose degrading enzymes called glucanases [19] and PD-localized proteins (PDLPs) [65]; 3) SCD1, as it plays a role in cytokinesis [69], and *scd1* seedling showed phenotype similar to *essp8* (See Additional file 2: Figure S5); and 4) GSL10, since GSL8 and GSL10 are the most closely-related members of the GSL family (clustered into the same subfamily) according to the phylogenetic tree for the Arabidopsis GSL family (See Additional file 2: Figure S8), and *gsl8* and *gsl10* loss-of-function mutants show similar phenotypes during microspore mitosis and sporophyte development [34]. We thus speculated that GSL8 and GSL10 might form a heterodimeric complex, in which the absence of one member would disrupt the function of the complex. Altogether, six candidates including SUCROSE SYNTHASE 1 (SUS1), UDP-GLYCOSYLTRANSFERASE (UDPG), PDLP5, a β-1,3-glucanase called AtBG_PPAP, SCD1 and GSL10 were selected to investigate their interaction with GSL8 in vivo. First, we performed bimolecular fluorescence complementation (BiFC) assays to test the interaction of AtBG_PPAP, PDLP5, UDPG, SCD1 and SUS1 with GSL8. All the five tested proteins showed an interaction with GSL8 *in planta* (Fig. 6a-e). Subcellular localization of GSL8-YFP fusion shows its localization at the cell membrane (Fig. 6f). No interaction was detected between GSL8-YC and GmIFS2-YN, an ER membrane-localized protein from soybean [70], and pEG100-YN which were used as negative control (Fig. 6g-h). The localization of the interacting proteins was also visualized by their YFP signals (See Additional file 2: Figure S9). Next, we used Förster resonance energy transfer (FRET) to further validate these interactions. FRET analysis showed the potential interaction between GSL8 and SUS1, AtBG_PPAP, PDLP5 and SCD1. No interaction could be detected between GSL8 and UDPG (Fig. 6i, See Additional file 2: Figure S10). Considering the higher sensitivity of FRET compared to BiFC, we suggest that GSL8 interaction with UDPG might occur indirectly through another protein. It needs to be noted that, regardless of our exhaustive attempts, propagation of the GSL10 cDNA in bacteria was not successful and thus its interaction with GSL8 could not be tested by BiFC and FRET. Lastly, to confirm the interactions identified by both BiFC and FRET, a membrane yeast two hybrid system (MYTH) [71] was employed. The MYTH results confirm the interaction of GSL8 with AtBG_PPAP, PDLP5, GSL10, SCD1 and SUS1 in yeast, albeit the interactions with SCD1 and SUS1 are rather weak (Fig. 6j).

The possibility that GSL8 and GSL10 interact to form a complex was further tested using a genetic approach. It was hypothesized that *gsl8 gsl10* double mutant shows a similar phenotype to that of *gsl8* or *gsl10* single mutants if GSL8 and GSL10 form a heterodimeric complex. Due to the essential role of GSL10 in gametophytic development, no homozygous *gsl10* mutants could be recovered after screening several T-DNA insertion lines. Hence, a conditional *gsl8 gsl10* double mutant was generated using an artificial miRNA under the control of an estradiol-inducible promoter [72]. The mock treated fourteen-day-old *XVE:aMIRGSL8/GSL10* transgenic seedlings did not show any obvious defects compared to the WT, whereas the transgenic seedlings treated with β-esteradiol phenocopied the *gsl8–1* phenotype and did not exhibit more severe defects compared to *gsl8* single mutant (See Additional file 2: Figure S11A-D). Analysis of the *GSL8* and *GSL10* transcript levels in *XVE:aMIRGSL8/GSL10* treated seedlings confirmed their downregulation compared to WT and mock-treated seedlings (See Additional file 2: Figure S11E). This result is consistent with a scenario that GSL8 and GSL10 are not functionally-redundant, rather, they become functional by forming a dimer which might be part of the callose synthase complex.

## Discussion

### Polyploidization as a cause of *gsl8* lethality

Callose is required for completing plant cytokinesis and proper cell wall formation [2, 8, 29, 73]. Previous reports have shown that loss-of-function mutations in *GSL8* cause defects in cell plate and cell wall formation in reproductive tissues [30, 33–36]. Our findings are in agreement with the previous studies and reconfirm the cytokinesis defects in the newly identified allele of *GSL8*, *essp8*. Furthermore, we showed that *gsl8* mutation induces ectopic polyploidization and endomitosis, both in the meristematic tissue and elongating cells in the primary root of the seedling. Our results provide evidence that cytokinesis defects in *gsl8* mutants are beyond the reproductive tissues and affect both somatic and reproductive cells.

The cause(s) of *gsl8* knockout mutants’ lethality is still unclear. Loss of proper chromosome condensation and segregation during successive cell divisions has been suggested as one of the potential reasons leading to growth arrest [35]. Segregation of the replicated chromosomes can become too complicated in polyploid or endomitotic nuclei as they go through consecutive cell divisions [74]. The significant increase in the number of polyploid and/or endomitotic cells in older *essp8* seedlings suggest that accumulative polyploidization caused by defects in cell plate formation might induce a premature arrest of cell division in proliferating tissues and, consequently, cause cell death. Death through mitotic catastrophe and polyploidization has been reported in a number of species [75–80].

### Controlled symplastic movement of SHR and miR165/6 requires GSL8

Symplastic signaling through PD is a dynamic process [81]. However, it is still not clear how callose synthases regulate PD and what other molecular components are required for this regulation. GSL8 was previously shown to be associated with PD regulation in leaf epidermal cells [30]. Expression analysis of *GSL8* indicated its high expression in the vasculature and actively dividing cells [32, 33]. In the vasculature, callose is mostly deposited at PD. A recent study suggested that an effective auxin gradient is established through GSL8-mediated callose deposition at PD, leading to downregulation of symplastic permeability [52]. Our results showed a significant decrease of callose accumulation in the primary root of all studied *gsl8* mutants. Correlating with a reduction of callose accumulation at PD, the cell-to-cell diffusion assay demonstrated that the *essp8* mutation results in an increase in symplastic movement in hypocotyls, which consequently permits passive diffusion. Our finding also indicates that PD defects in *gsl8* mutants are not restricted to epidermal cells, as was previously documented in *chorus* [30].

Our work reveals that SHR and miR165/6 distribution patterns are altered in *gsl8* mutants and provides a molecular explanation for their root phenotype. SHR is required for cell division and endodermis differentiation [53, 56, 82, 83]. Ectopic movement of SHR induces an increase in the number of cell layers, where the cells exhibit endodermal characteristics [83–85]. SHR abundance changes dynamically during root development, and its dose regulates middle cortex formation and periclinal cell division. High levels of SHR in the endodermal cells inhibit periclinal cell division [86]. Here, we provide new evidence that SHR cell-to-cell movement is dysregulated in *essp8* mutants as shown by an increase in endodermal SHR-GFP relative to the WT (Fig. 4I). Consistent with a previous finding [86], we observed loss of periclinal cell division and middle cortex formation in ten-day-old seedlings (See Additional file 2: Figure S3). As *essp8* seedling ages, root tissue layers become more disorganized. Therefore, we conclude that the *essp8* root phenotype is likely to be caused, at least in part, by dysregulation of SHR symplastic movement.

SHR directly binds to the 5′ upstream regions of *MIR165A* and *MIR166B* to activate their transcription [61]. Mature miR165/6 will move from endodermis to stele where they suppress the expression of *HD-ZIP III* family genes [87, 88]. Our analysis revealed that the movement of mature miR165/6 is dysregulated in primary root of *gsl8* mutants, suggesting that GSL8-mediated callose deposition at PD is required for regulation of miR165/6 trafficking. Importantly, the defects in vasculature tissue patterning in *gsl8* mutants are reminiscent of the phenotype of *MIR165/6* overexpression lines and *HD-ZIPIII* quadruple mutants [61]. Hence, we suggest that ectopic activity of miR165/6 in the primary root of *essp8* could be, at least partially, responsible for the *essp8* root phenotype. However, as the SHR/SCR complex regulates *MIR165/6* expression [61, 62], it still needs to be further investigated whether the elevated miR165/6 activity in *gsl8* is a direct effect of an increase in its bidirectional PD-mediated movement, or is due to the elevated level of SHR in the endodermal cells, or is caused by both. It is worth noting that our observation with *gsl8* loss-of-function mutants is complementary to a previous study showing restriction of SHR and miR165/6 PD-mediated movement in a *gsl12* gain-of-function mutant [29]. Our data support the notion that GSL8-mediated callose deposition at PD is required for regulation of cell-to-cell communication during early seedling development. Although it is challenging to discriminate the defects caused by cytokinesis impairment and loss of callose deposition at the PD, we showed that PD biogenesis is unlikely to be affected in *essp8* mutants. We conclude that dysregulation of symplastic trafficking is due to the reduced amount of callose deposited at the PD and, consequently, PD relaxation.

Two other members of the Arabidopsis GSL family, GSL7 and GSL12, were shown to be involved in callose biosynthesis at the PD [29, 89, 90]. *GSL7* is expressed in the vasculature system and is required only for callose deposition at the phloem PD and sieve plates [31, 91]. *gsl7* mutants do not display any obvious macroscopic phenotypic defects, suggesting that GSL7 has no biological function other than phloem-specific PD callose synthesis. Vaten et al. (2011) suggested a role for GSL12 in PD regulation using an inducible expression system for *GSL12* gain-of-function mutants [19, 29]. They showed that gain of function mutations or ectopic expression of *GSL12* leads to callose accumulation and plasmodesmatal connectivity decrease in the root [29]. The lethality of *essp8* single mutant rules out the possibility of GSL8 and GSL12 being functionally redundant; however, it suggests that the GSL enzymes have similar functions. Taken together, previous studies and our current data indicate that the spatial regulation of GSLs and their function play critical role in regulating plasmodesmal function during early seedling development in Arabidopsis.

### A multi-subunit CalS complex in plasmodesmata aperture regulation

Callose biosynthesis and its deposition need to be highly-regulated. It has been proposed that GSLs, e.g. GSL8, are integrated into an extremely specialized protein complex to carry out callose synthesis. The mechanisms employed by β-1,3-glucanases, for degrading callose, and by PDLPs, for inducing callose deposition at PD, have remained unclear. Based on our observations, here we propose that regulation of the callose level at PD and the balance between the activities of enzymes synthesizing and degrading callose occurs through the direct interaction of GSL8 and AtBG_PPAP. Furthermore, as was previously proposed [20], PDLP5 is likely to induce and modulate callose deposition at PD through its direct interaction with a callose synthesis enzyme. A recent study suggested that callose deposition at PD by GSL8 occurs through an independent pathway from PDLP5 [28]. Here, we propose that callose induction at PD by PDLP5 is (likely) to be GSL8-mediated.

It was previously suggested that UGT1 transfers UDP-glucose (UDP-Glc) from SuSy to CalS, and therefore, channels callose deposition to the targeted subcellular location [67, 68]. GSL8 interaction with SUS1 and UDPG was shown to occur in the cytoplasm and on the endoplasmic reticulum (ER) in BiFC assay; however, GSL8 interaction with UDPG was not confirmed by FRET. Considering the subcellular localization of these proteins (See Additional file 2: Figure S9), our data lead us to speculate that UDPG interaction with GSL8 might be indirect. Thus, similar to the case of the cellulose synthase (CESA) complex [92, 93], sucrose synthase is likely to be incorporated into the callose synthase complex to channel UDP-Glc into glucan synthesis. Obviously, other components of sucrose degradation and biosynthesis pathway should be tested to identify other related players that directly interact with GSL8.

A recent study has shown that SCD proteins, including SCD1, are involved in different membrane trafficking events required not only for cytokinesis, but also for cell expansion [69]. During cell plate formation, SCD1 is involved in vesicular trafficking to the equator of the dividing cell [50]. Callose deposition converts the fused vesicles into the plate [73]. The GSL8-SCD1 interaction was detected at the cell membrane in BiFC and FRET, and showed weak interaction in MYTH. This finding indicates that there is a GSL8-SCD1 interplay. It is already known that secondary PD are formed post-cytokinesis, possibly during cell expansion; however, how this happens remains unclear [94]. Future investigations are needed to elucidate whether the GSL8-SCD1 interplay is restricted to cell plate formation and/or is somehow linked to secondary PD formation.

GSL10 has remained as one of the least studied members of the GSL family due to severe gametophyte defects in the mutant lines. A previous study speculated the existence of a GSL8-GSL10 heterodimeric callose synthase-like complex [34]. Our observations support this hypothesis based on our MYTH result (Fig. 6j) and the observation with the *gsl8 gsl10* conditional double mutant (See Additional file 2: Figure S11). Future study on co-localization of GSL8 and GSL10 will provide further evidence on existence of such a complex. Similar heteromeric complex have been shown for CESA complexes formed by three different CESA proteins [95, 96].

## Conclusion

**Fig. 7:**
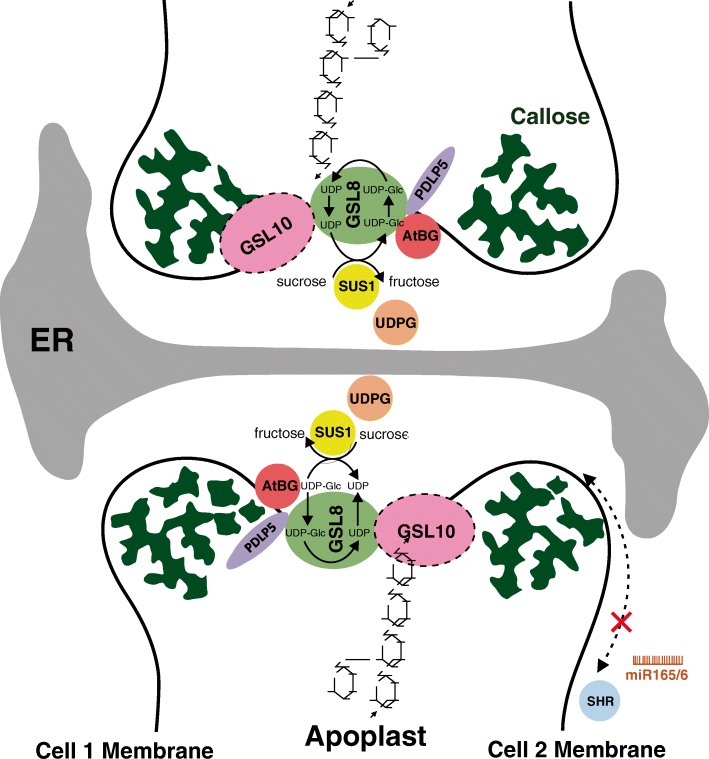
Proposed model of callose deposition regulation at plasmodesmata. GSL8 (possibly GSL10 as well) catalyzes the synthesis of callose that is deposited at the neck of plasmodesmata. UDP-Glc is the callose precursor that is released from sucrose hydrolysis by SUS1 with the help of UDPG, and then channeled to GSL8 through the GSL8-SUS1 physical interaction. Conversely, there is another family of proteins including AtBG_PPAP that degrade callose. The GSL8-AtBG interaction observed in this work suggests a direct synthesizer-degrader interaction to control the deposition level of callose at plasmodesmata through an as yet unknown mechanism, which might involve PDLP5. This highly regulated callose deposition at plasmodesmata allows the proper symplastic movement of SHR and miR165/6 during development

Our current study reports the critical role of GSL8 during early seedling development in Arabidopsis. We show how callose biosynthesis by GSL8 is required to complete cytokinesis during cell division, and to regulate cell-to-cell communication. Callose accumulation occurs through interaction of different components, likely to be incorporated into a callose biosynthesis complex to highly regulate callose deposition at the PD. Our observations, for the first time, provide new evidence supporting an earlier hypothesis that GSL8 might have regulatory roles apart from its enzymatic function in PD regulation (see model in Fig. 7). Further studies along these lines will probably result in dissection of the other components that mediate callose biosynthesis at the PD and control PD SEL.

## Methods

### Plant material

Due to the lethality of *gsl8* mutants, all lines were propagated as heterozygotes. *essp8* was identified in a screen of EMS-mutagenized population [37]. Seeds for WT Col-0, L*er*, and different T-DNA lines used in this study (See Additional file 1: Table S4) were ordered from Arabidopsis Biological Resource Center (ABRC).

### Next-generation mapping of *essp8*

For genetic mapping of the *essp8* mutation, M2 plants from a Col-0 background were crossed with WT plants of the L*er* accession. A total of 100 two-week-old seedlings with *essp8* dwarf phenotype were selected from the F2 segregating population and used for bulked-segregant analysis (BSA) and rough-mapping. Pooled genomic DNA (gDNA) was used for BSA with 22 pairs of simple sequence length polymorphism (SSLP) markers [97]. The genomic interval harbouring the *essp8* mutation was narrowed down using PCR-based rough-mapping (See Additional file 1: Table S5). Pooled gDNA extracted from 64 seedlings were used as template for next-generation sequencing (NGS) [98]. The NGS library was generated using NGS library preparation kit (Zymo Research). Sequencing was performed on the Illumina MiSeq (Illumina, USA).

### Histochemical assays

For callose staining, a stock solution of 0.1 mg/ml aniline blue fluorochrome (Biosupplies Australia PTY Ltd.) was prepared in distilled water. Prior to use, the stock solution was diluted 1:3 with 0.1 M K_3_PO_4_, pH 12.0. Roots of seven-day-old Arabidopsis seedlings were incubated with fluorochrome staining solution for 30 min then washed with 0.1 M K_3_PO_4_, pH 12.0 buffer and imaged on a Zeiss Axioscope 2 (Zeiss, Germany) compound fluorescence microscope using a UV laser. The microscope was integrated with a Nikon DS-Ri2 digital camera using the ACT-1 software (Nikon, Japan).

To stain the cell walls with PI [99], Arabidopsis roots were immersed in 1 μg/ml solution for 3 min. To visualize both cell walls and nuclei, 100 μg/ml PI solution was used. Roots were stained for at least 5 min and rinsed twice with distilled water. PI-stained roots were imaged on a Leica TCS SP2 Laser Scanning confocal microscope (Leica, Germany) using 543 nm excitation and 610–630 nm emission.

For PD SEL assay, seeds were allowed to germinate in the dark for seven days. Dextran, Alexa Fluor® 488; 3,000 MW, Anionic (ThermoFisher Scientific) and Dextran, fluorescein, 10,000 MW, Anionic (ThermoFisher Scientific) were dissolved in Tris-EDTA buffer, pH 8.0 at concentrations of 100 mg/ml and 50 mg/ml, respectively. Prior to use, the stocks were diluted in Tris-EDTA buffer at a ratio of 1:10. The hypocotyls were obtained by cutting the seedlings at the hook. For each sample, 1 μl of the diluted probe was injected into the hypocotyl at the cut site using a Hamilton Gastight syringe (Hamilton). Movement of the probe was analyzed immediately by imaging on a Leica TCS SP2 Laser Scanning confocal microscope (Leica) using 488 nm excitation and 515 to 530 nm emissions.

### Generation of transgenic plants

Except from the complementation, the rest of the transgene constructs were generated using the Gateway™ system (Invitrogen) [100]. Floral dipping of plants were carried out as described previously [101].

To complement *essp8*, the translational cassette for *GSL8* was generated by first, synthesizing the 2.7 kb sequence upstream of the Start codon harboring the native promoter, the 5′ untranslated region (UTR), followed by *GSL8* coding sequence (BIO BASIC Int.). The synthesized cassette was subcloned into the modified yeast-compatible pGREEN vector using TAR-cloning [102] through homologous recombination in yeast [103].

To create the *SHR-GFP* translational fusion construct, a 3 kb genomic fragment harbouring the promoter, the 5’ UTR, and the genomic sequence (not including the STOP codon) was amplified from Arabidopsis gDNA (See Additional file 1: Table S6), and cloned into the pMDC107 vector [72]. The construct was transformed into plants by floral dipping [101]. Transgenic *pSHR:SHR-GFP* plants were selected on MS agar media containing 50 μg/ml hygromycin B. The PDLP5-GFP expression construct (*p35S:PDLP5-GFP*) was generated by amplifying the cDNA and cloning it into the pEarleyGate103 [104] using the Gateway™ system (Invitrogen) [100].

Transgenic seeds expressing the centromere labeling construct (*p35S:CENH3-GFP*) were described previously [35]. To visualize the centromeres in *gsl8* mutants, plants homozygous for *p35S:CENH3-GFP* were crossed with *GSL8/essp8*, *GSL8/gsl8–2* and *GSL8/gsl8–4* plants. The F2 plants were used for imaging and quantifying the number of centromeres.

To test the miR165/6 activity in *gsl8* mutants, previously described miR156/6 sensor line [61] was crossed with heterozygous *GSL8/gsl8* plants and F2 seedlings were used for analysis and imaging.

The Web Micro Designer (WMD, http://wmd3.weigelworld.org/cgi-bin/webapp.cgi) was used for designing artificial miRNAs (amiRNAs) against both *GSL8* and *GSL10* genes. To generate the *XVE:aMIRGSL8/GSL10* construct, first the amiRNA sequence was introduced into the pRS300 vector [105] as the backbone to create *aMIRGSL8/GSL10* which was then subcloned into the donor vector pDONR221 (Invitrogen). The donor construct was then recombined into the pMDC7 Gateway-compatible destination vector [72]. In pMDC7, the *aMIRGSL8/GSL10* transgene is controlled by an estradiol-inducible promoter. WT Col-0 plants were transformed with the construct by floral dipping [101]. Transgenic plants were selected on MS agar media containing hygromycin B. T2 transgenic seeds were sown on MS agar media containing 100 μM β–estradiol or dimethyl sulfoxide (DMSO) as a mock control.

### Gene expression analysis

For quantitative reverse transcription-PCR (qRT-PCR), total RNA was isolated from ~ 100 mg of plant tissue using the RNeasy Mini Kit (Qiagen). The High Capacity cDNA Reverse Transcription kit (ABI) was used to reverse transcribe total RNA into cDNA with random primers from the kit. qRT-PCR was performed using the SsoFast EvaGreen Supermix kit (Bio-Rad Laboratories, Inc.) with the Bio-Rad CFX96 real-time PCR detection system (Bio-Rad Laboratories, Inc. USA). The data shown are the average of three technical and three biological replicates. *GLYCERALDEHYDE-3-PHOSPHATE DEHYDROGENASE* (*GAPDH*) was used as the internal reference [106]. PCR primers used in qRT-PCR are listed in Additional file 1: Table S6.

### Bimolecular fluorescent complementation

Full-length cDNA was used for the BiFC experiment. Amplified cDNA was cloned into pEarlyGate100-YN and pEarlyGate100-YC [38, 107] using the Gateway system (Invitrogen). Four-week-old *N. bentamiana* leaves were infiltrated as described previously [108] with two constructs expressing GSL8-YC and the candidate interactors fused to YN. Three days post infiltration (dpi), the fluorescent cells' images were captured on a Leica TCS SP2 Laser Scanning confocal microscope (Leica) [109] using 514 nm excitation and 515 to 545 nm emission. Primers used in generation of BiFC constructs are listed in Additional file 1: Table S6.

### Förster resonance energy transfer

To test the interaction of GSL8 with candidate partners, their full-length cDNA was amplified from Arabidopsis cDNA pool and cloned into pEarleyGate101 and pEarleyGate102 [104] using the Gateway system (Invitrogen) to generate YFP- and CFP-fusion proteins, respectively (see Additional file 1: Table S6). The infiltration was performed as described in BiFC with two constructs expressing GSL8-YFP and the candidate interactor fused to CFP. The FRET between two proteins was quantified 3dpi using acceptor photobleaching method by imaging on a Leica TCS SP2 Laser Scanning confocal microscope (Leica) [109]. Images of the CFP fluorescence, for donor protein, and YFP fluorescence for acceptor proteins were captured using 458 nm excitation and 465 to 505 nm emissions, and 514 nm excitation and 525 to 600 nm emission, respectively. The fluorescence of the CFP and YFP channels were scanned before and after bleaching. Bleaching of the acceptor protein fluorescence was performed using 100% excitation of 514 nm beam for 50 frames. The energy transfer efficiency between the two protein pairs was measured based on the fluorescence intensity change in the donor and acceptor, before and after photobleaching. Three independent experiments with at least three biological replicates for each pair were used to calculate FRET efficiency.

### Membrane yeast two-hybrid

The MYTH system was used as described by Snider et al. [71]. Prey constructs were cloned in the pPR3-C and bait construct was cloned in the pAMBV vectors. The cDNA were cloned by ‘gap-repair’ homologous recombination in yeast [110]. After co-transformation of bait and prey vectors, presence of interaction was analysed by comparing colony growth on transformation selection media (TSM) (SD-Leu-Trp) and interaction selection media (ISM) (SD-Leu-Trp-Ade-His).

### Microscopy and image analysis

Images were captured by a Nikon SMZ1500 (Nikon) dissecting or Zeiss Axioscope 2 (Zeiss) compound light microscopes which were integrated with a Nikon DS-Ri2 digital camera using the ACT-1 software (Nikon). Nikon dissecting scope optical ranges varied between 0.75 and 11.5X, and the compound Zeiss microscope was used with 20 and 40X objectives. TIFF format at a resolution of 3840 × 3072 pixels was used for capturing all images. The ImageJ software [111] was used to quantify callose and measuring GFP intensity levels on unmodified root images. Callose quantification at the PD was carried out as previously described [112]. The endodermal-to-stele ratio of SHR-GFP was measured in the WT and *gsl8* mutants as earlier described [59]. Briefly, the level of SHR-GFP fluorescence was measured in the endodermis and expressed as a ratio of stele fluorescence. To determine the level of fluorescence in each given region, e.g. endodermis or stele, the corrected total cell fluorescence (CTCF) was calculated using the following formula: CTCF = Integrated Density - (Area of selected cell X Mean fluorescence of background readings) [113–115]. The average for the WT control was set to 1, and the intensity ratio in the mutants was calculated relative to the WT. Statistics were done using Excel (Microsoft Office). A minimum of 10 and maximum of 20 roots were used for both callose intensity and SHR-GFP quantification analysis.

### Accession numbers

Sequence data used in this article can be found on the Arabidopsis Information Resource (TAIR) database under the following accession numbers: *GSL1* (AT4G04970), *GSL2* (AT2G13680), *GSL3* (AT2G31960), *GSL4* (AT3G14570), *GSL5* (AT4G03550), *GSL6* (AT1G05570), *GSL7* (AT1G06490), *GSL8* (AT2G36850), *GSL9* (AT5G36870), *GSL10* (AT3G07160), *GSL11* (AT3G59100), *GSL12* (AT5G13000), *HINKEL* (AT1G18370), *KNOLLE* (At1G08560), *KEULE* (AT1G12360), *KORRIGAN* (AT5G49720), *SCD1* (AT1G49040), *SHR* (AT4G37650), *SCR* (AT3G54220), *UDPG* (AT1G16570), *SUS1* (AT5G20830), *AtBG_PPAP* (At5G42100), *PDLP5* (AT1G70690).

## Additional files

Additional file 1 **Figure S1.** Mapping of the *essp8* mutation. **Figure S2.** *ESSP8* is an allele of *GSL8*. **Figure S3.** Morphological phenotype of *gsl8* mutants’ primary root showing severe defects in root tissue patterning. **Figure S4**. Genetic complementation of *essp8* seedlings. **Figure S5.** Morphological phenotype of cytokinesis-defective mutants. **Figure S6.** Movement of fluorescent probes in *essp8* hypocotyls. **Figure S7.** *SCR* relative expression in *essp8* primary root compared to WT. **Figure S8.** Phylogenetic tree of Arabidopsis GSLs. **Figure S9.** Subcellular localization of the putative callose synthase complex components using their YFP fusions in transiently transformed *N. bentamiana* epithelial cell. **Figure S10.** FRET assay: Images of CFP and YFP fluorescent before and after bleaching. **Figure S11.** Morphological phenotype of the *XVE:aMIRGSL8/GSL10* seedlings. (PDF 10100 kb)

Additional file 2 **Table S1.** Segregation of homozygous *essp8* seedlings in the progeny of selfed *ESSP8/essp8* heterozygous plants. **Table S2.** The percentage of defective seeds in one silique from selfed *ESSP8/essp8* heterozygous plants. **Table S3.** Segregation of homozygous *gsl8* T-DNA insertion seedlings in the progeny of selfed heterozygous plants. **Table S4.** List of mutant lines and primers used for genotyping. **Table S5.** List of primers used for *essp8* rough-mapping. **Table S6.** List of primers used for cloning and qPCR. (PDF 210 kb)

## Abbreviations

Cals: Callose synthase
dpi: Days post-infiltration
GFP: Green fluorescent protein
GSL: Glucan synthase
PD: Plasmodesmata
SEL: Size exclusion limit
