# Supplementary material for: Analysis of a novel mutant allele of GSL8 reveals its key roles in cytokinesis and symplastic trafficking in Arabidopsis
Source: BMC Plant Biol. 2018 Nov 22;18:295. doi: 10.1186/s12870-018-1515-y (PMC6249969; doi:10.1186/s12870-018-1515-y)
Supplement: Supplementary file 1 — Figure S1. Mapping of the essp8 mutation. Figure S2. ESSP8 is an allele of GSL8. Figure S3. Morphological phenotype of gsl8 mutants’ primary root showing severe defects in root tissue patterning. Figure S4. Genetic complementation of essp8 seedlings. Figure S5. Morphological phenotype of cytokinesis-defective mutants. Figure S6. Movement of fluorescent probes in essp8 hypocotyls. Figure S7. SCR relative expression in essp8 primary root compared to WT. Figure S8. Phylogenetic tree of Arabidopsis GSLs. Figure S9. Subcellular localization of the putative callose synthase complex components using their YFP fusions in transiently transformed N. bentamiana epithelial cell. Figure S10. FRET assay: Images of CFP and YFP fluorescent before and after bleaching. Figure S11. Morphological phenotype of the XVE:aMIRGSL8/GSL10 seedlings. (PDF 10100 kb) [file 12870_2018_1515_MOESM1_ESM.pdf]

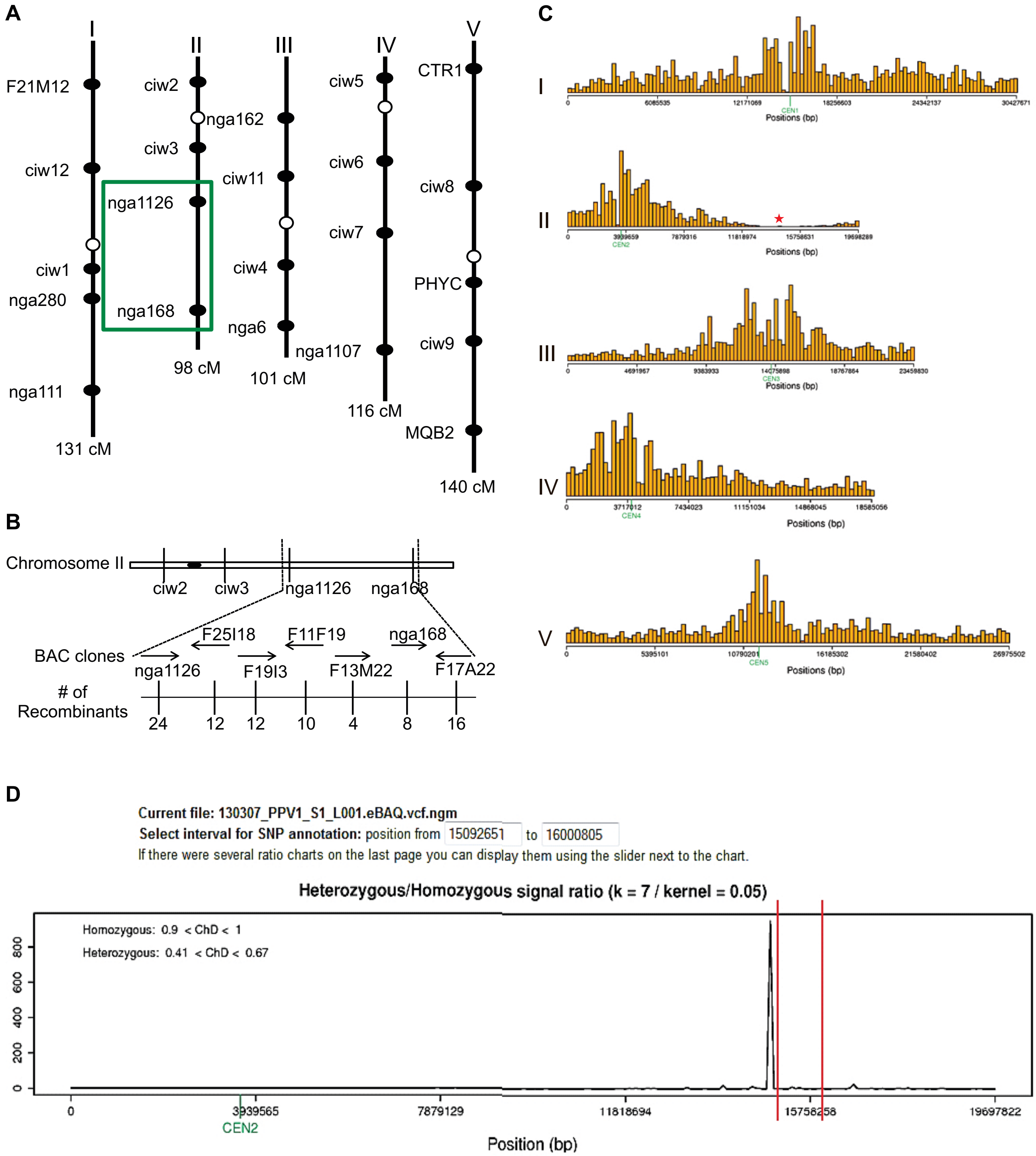

SNP annotations:

Chrom.	Position	Ref. base	SNP base	Depth	Discordant chastity	Accession	Tag	Strand	Ref. codon	SNP codon	AA change	BLOSUM 100
2	15463115	C	T	2	1.00	AT2G36850.1	cryptic splice site (1bp)					

Fig. S1

Figure S1. Mapping of the *essp8* mutation.

(A) Using bulked-segregant analysis (BSA), the *essp8* mutation was located on the bottom arm of chromosome 2 between the nga1126 and nga168 markers (green box). (B) The position of the *essp8* mutation was narrowed down to a genomic interval of 729 kb, covered by the BAC clones F11F19 and F13M22, on chromosome II. The number of recombination events out of the total number of chromosomes examined (200) are shown. (C) NGM (Next Generation Mapping) revealed one SNP desert region on the bottom arm of chromosome II (red star). The bars represent the natural polymorphism between wild-type Col-0 and L*er* accessions. (D) The combination of rough mapping and NGM results in the identification of one SNP caused by EMS within the candidate region (between the red lines) on chromosome II. The GC to AT substitution is located at an intron splice site of AT2G36850 (*GSL8*).

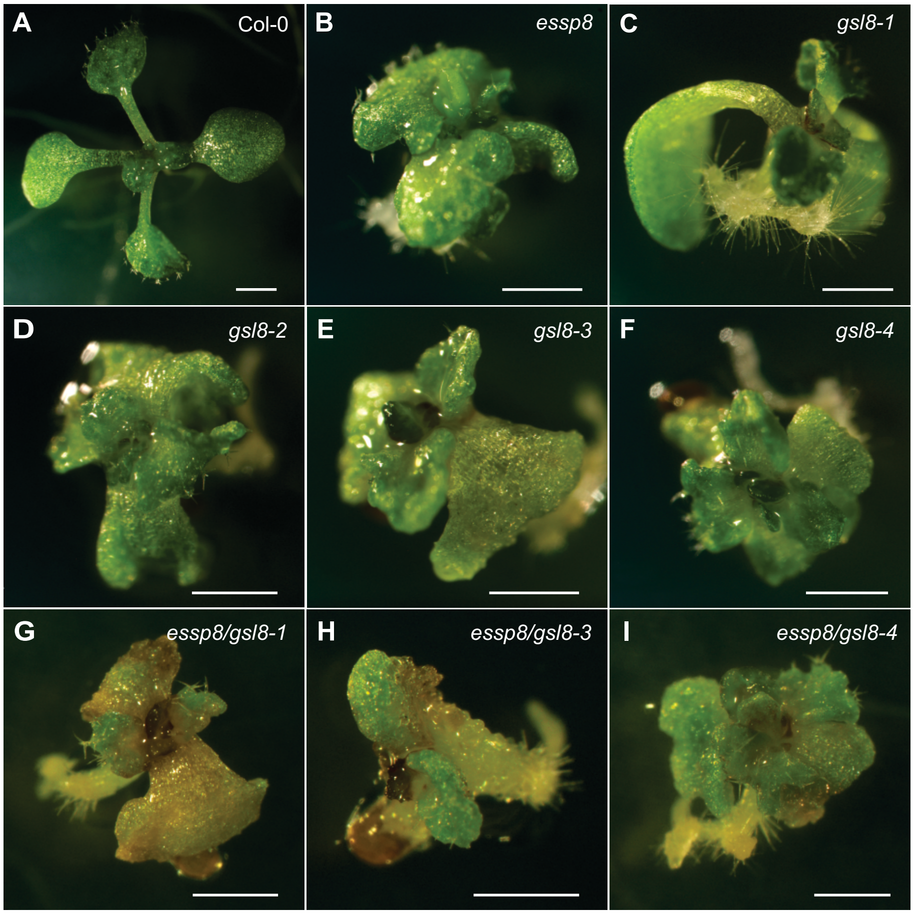

Figure S2. *ESSP8* is an allele of *GSL8*.

(A-F) Morphological phenotype of ten-day-old seedlings of *essp8* and *gsl8* T-DNA insertion mutants. (G-I) Morphological phenotype of ten-day-old F_1 seedlings carrying two different alleles of *GSL8.* Seedlings that were heterozygous for two different mutant alleles of *GSL8*, i.e., *essp8* with *gsl8-1* (G), *gsl8-3* (H), or *gsl8-4* (I), respectively, exhibited similar phenotype to that of homozygous *gsl8* mutants. Scale bars = 1 mm

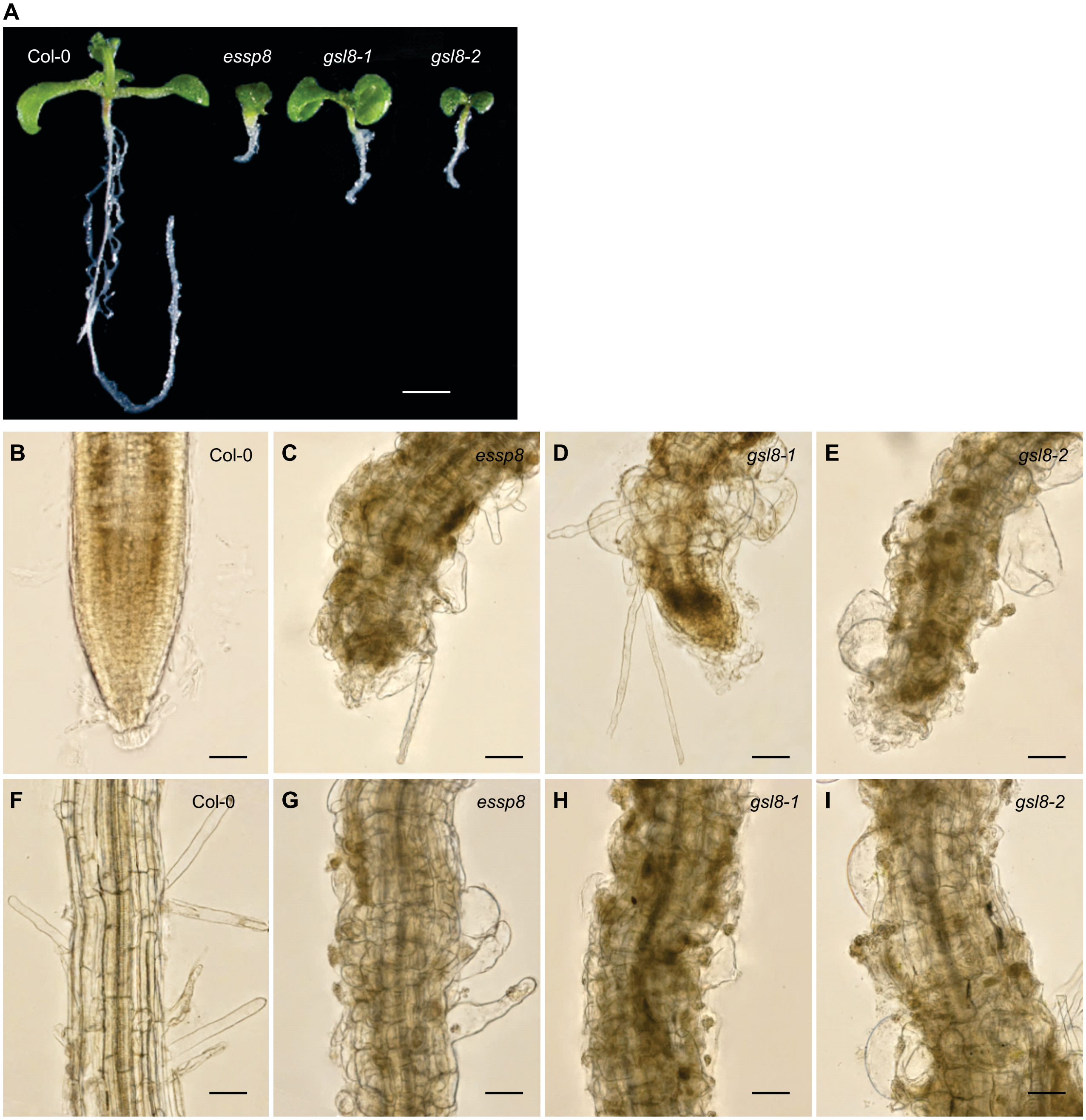
A
Col-0
essp8
gsl8-1
gsl8-2
B
Col-0
C
essp8
D
gsl8-1
E
gsl8-2
F
Col-0
G
essp8
H
gsl8-1
I
gsl8-2

Fig. S3

Figure S3. Morphological phenotype of *gsl8* mutants' primary root showing severe defects in root tissue patterning.

(A) Ten-day-old *gls8* mutant seedlings (*essp8*, *gsl8-1,* and *gsl8-2*) develop stunted roots compared to the WT. (B-I) Comparison of the root phenotype between five-day-old WT and *gsl8* mutants at the root tip and elongation zone of the root. (C-E). Abnormally-developed root tips with bloated cells in *gsl8* mutants. (G-I) Short, swollen and often branched root hairs formed in elongation zone of the primary root in *gsl8* mutants. Note: A part of Fig. 1 has been reused in this figure for the purpose of comparison. Scale bars = 200 µm (A), 50 µm (B-I)

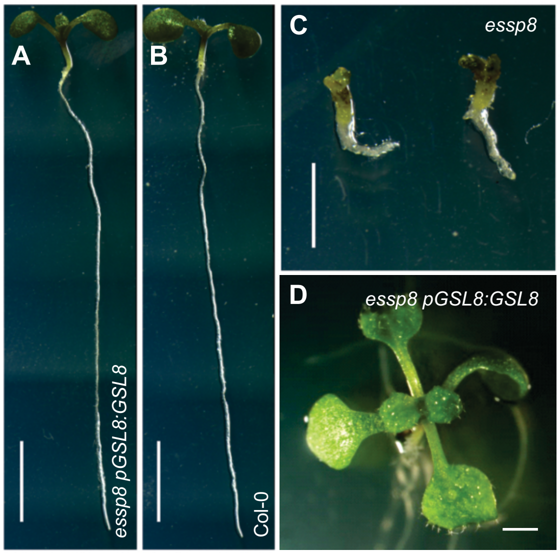

Figure S4. Genetic complementation of *essp8* seedlings.

(A-D) Wild-type *GSL8* rescues the root phenotype of *essp8* (A) as *essp8* seedling shows similar phenotype to the WT (B and D). *essp8* two-week old seedlings are shown as control (C).Scale bars= 1cm (A, B and D), 0.5cm (C)

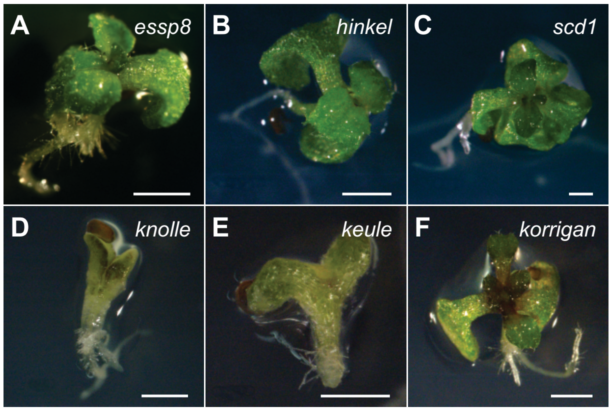

Figure S5. Morphological phenotype of cytokinesis-defective mutants.

(A-F) Comparison of *essp8* (A) with cytokinesis-defective mutant seedlings, *hinkel* (B), *scd1* (C), *knolle* (D), *keule* (E) and *korrigan* (F). All cytokinesis-defective mutants are dwarf, but *hinkel* and *scd1* are morphologically more similar to *essp8* with abnormally-developed cotyledons and true leaves (B), and thicker roots and hypocotyls (C), respectively. Scale bars = 1 mm

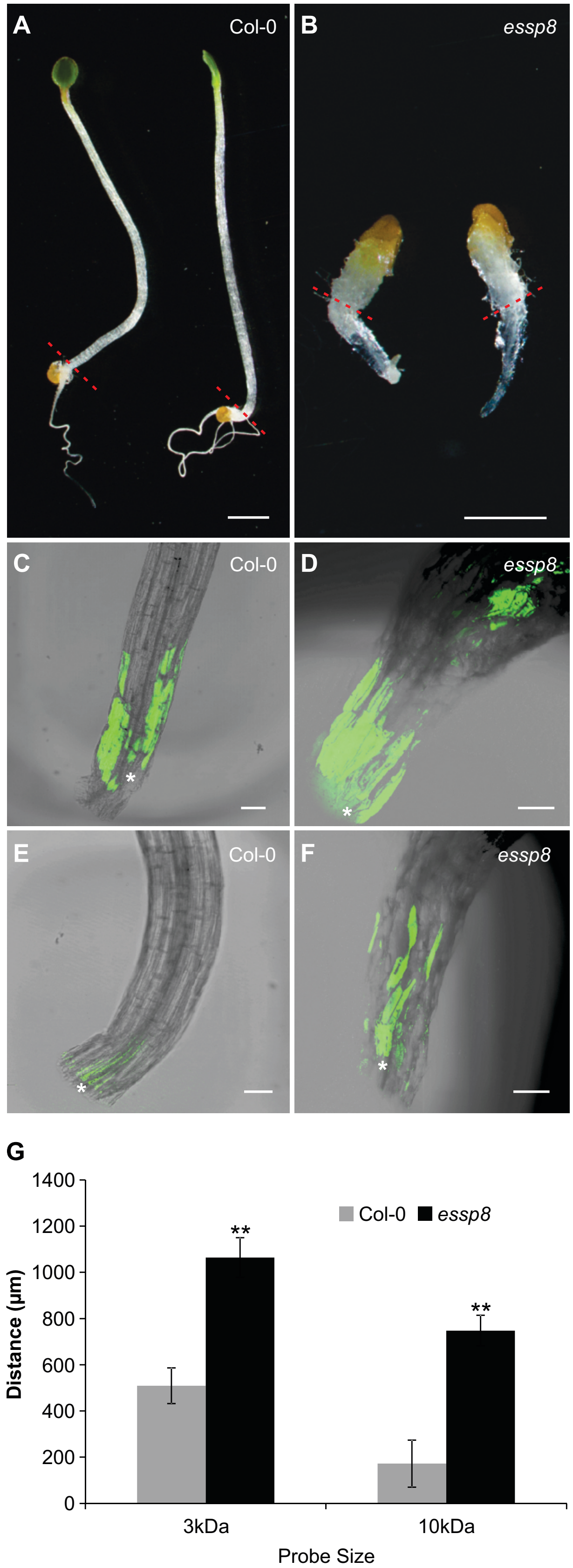
A
Col-0
B
essp8
C
Col-0
D
essp8
E
Col-0
F
essp8
G
1400
1200
1000
800
600
400
200
0
Distance (µm)
Col-0
essp8
**
**
3kDa
10kDa
Probe Size

Fig. S6

Fig. S6. Movement of fluorescent probes in *essp8* hypocotyls.

(A-B) The hypocotyls from WT (A) and *essp8* (B) etiolated seedlings were used for the assay. The dotted red lines indicate the position of cut sites. (C-F) Using two fluorescent probes, Alexa Fluor (3 kDa) (C-D) and fluorescein (10 kDa) (E-F), the symplastic connectivity was quantitatively measured in WT (C and E) and *essp8* (D and F) hypocotyls. The injection sites are shown by white asterisk. (G) Quantification of the fluorescent probes movement. In all cases, values reported are the mean ± SEM (n = 5). The double asterisks denote significant differences (Student's t-Test, ***P* < 0.01). Scale bars= 1 mm (A-B), 100 μm (C-F)

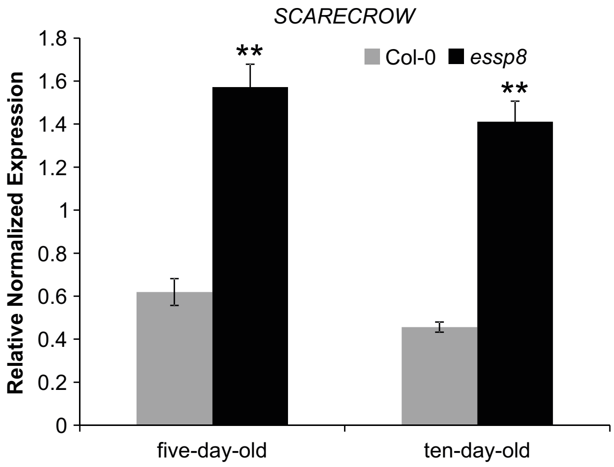

Figure S7. *SCR* relative expression in five-day-old and ten-day-old *essp8* primary root compared to WT. The expression level was normalized to that of *GAPDH*. Error bars indicate SEM of three technical and biological replicates. The double asterisks denote significant differences using Student's t-Test, **$P < 0.01$.

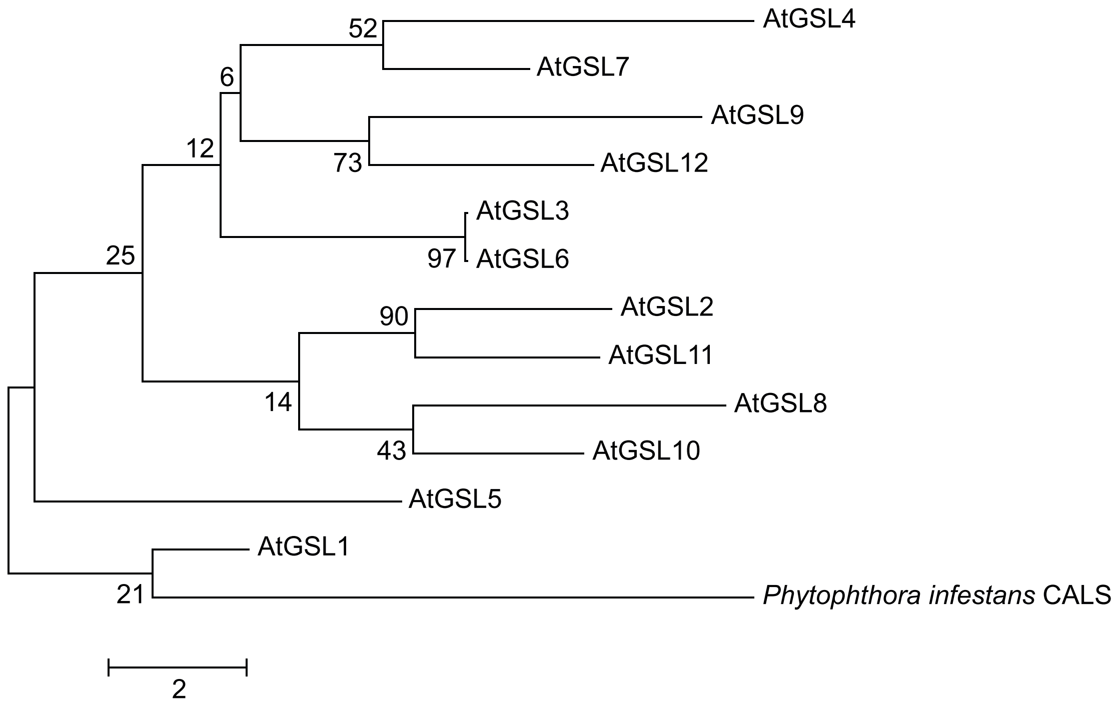

Figure S8. Phylogenetic tree of Arabidopsis GSLs.

The unrooted tree is based on an amino acid alignment of full-length sequences from Arabidopsis. The evolutionary history was inferred by using the maximum likelihood method. The tree with the highest log likelihood (-62692.0592) is shown. The percentage of trees in which the associated taxa clustered together is shown next to the branch points. The phylogenetic tree was generated with MEGA6 using a bootstrap value of 500.

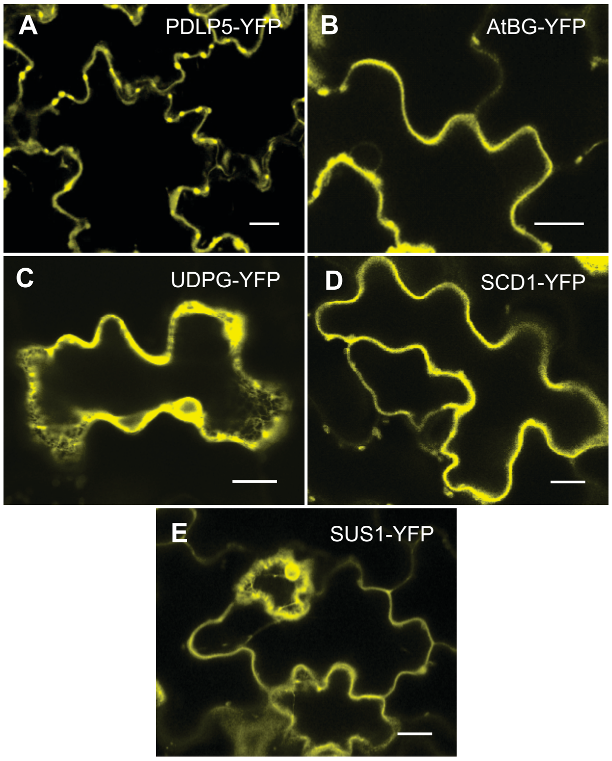

Figure S9. Subcellular localization of the putative callose synthase complex components using their YFP fusions in transiently transformed *N. bentamiana* epithelial cell. (A-B) Localization of PDLP5 and AtBG_PPAP to cell membrane and PD. (C) Localization of UDPG to cytoplasm and ER. (D) Localization of SCD1 to cell membrane. (E) Localization of SUS1 to cytoplasm and ER. Scale bars = 20 μm

Fig. S9

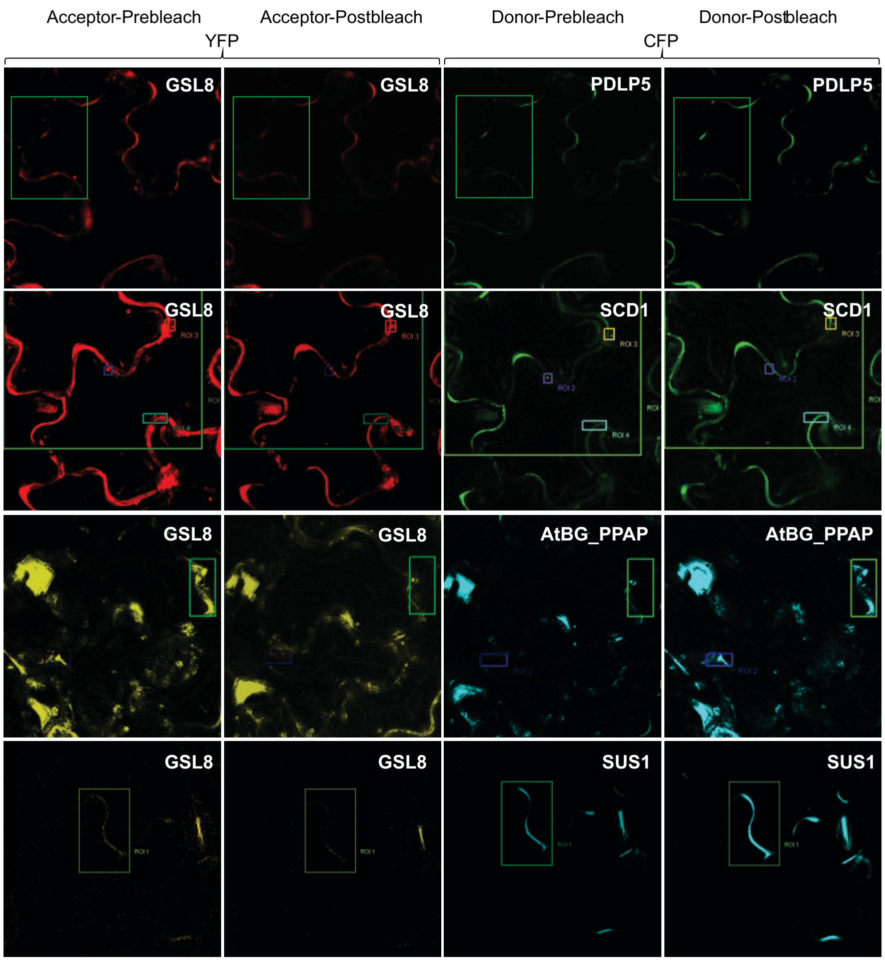

Figure S10. FRET assay. Images of CFP and YFP fluorescent before and after bleaching. Note weaker YFP fluorescence after bleaching, and an increase in CFP fluorescence in PDLP5, SCD1, AtBG_PPAP and SUS1.

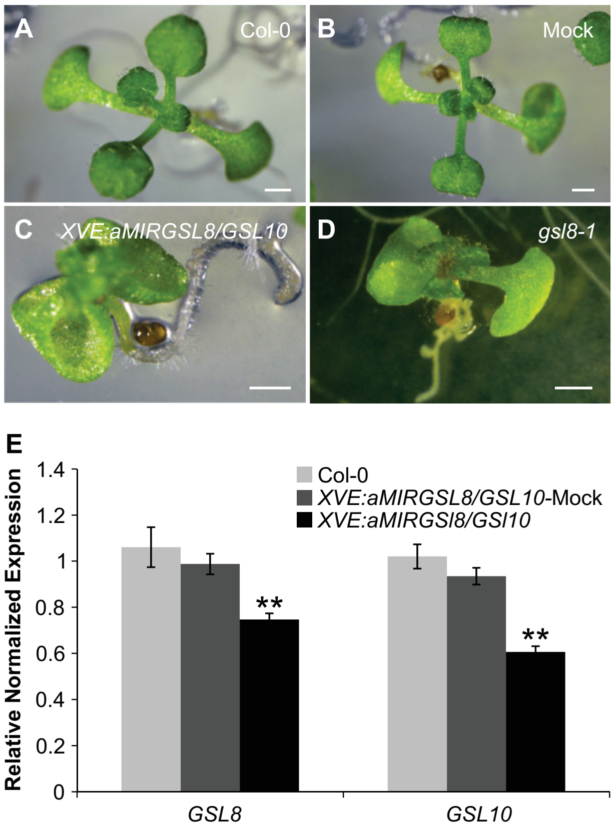

Figure S11. (A-D) Morphological phenotype of the *XVE:aMIRGSL8/GSL10* seedlings. Two-week-old WT (A), mock-treated *XVE:aMIRGSL8/GSL10* (B), induced *XVE:aMIRGSL8/GSL10* (C), and *gsl8-1* (D) seedlings. *XVE:aMIRGSL8/GSL10* transgenic seedlings phenocopy the *gsl8-1* mutant, dwarf with short and thick root and abnormally-shaped true leaves. Scale bars = 1 mm

(E) *GSL8 and GSL10* relative expression in two-week-old *XVE:aMIRGSL8/GSL10* transgenic seedlings mock treated or treated with 100 μm β-estradiol compared to WT. The expression of each one was normalized to that of *GAPDH*. Values represent means ± SEM of three technical and biological replicates. $^{**}P < 0.01$.
