# Supplementary material for: Analysis of a novel mutant allele of GSL8 reveals its key roles in cytokinesis and symplastic trafficking in Arabidopsis
Source: BMC Plant Biol. 2018 Nov 22;18:295. doi: 10.1186/s12870-018-1515-y (PMC6249969; doi:10.1186/s12870-018-1515-y)
Supplement: Supplementary file 2 — Table S1. Segregation of homozygous essp8 seedlings in the progeny of selfed ESSP8/essp8 heterozygous plants. Table S2. The percentage of defective seeds in one silique from selfed ESSP8/essp8 heterozygous plants. Table S3. Segregation of homozygous gsl8 T-DNA insertion seedlings in the progeny of selfed heterozygous plants. Table S4. List of mutant lines and primers used for genotyping. Table S5. List of primers used for essp8 rough-mapping. Table S6. List of primers used for cloning and qPCR. (PDF 210 kb) [file 12870_2018_1515_MOESM2_ESM.pdf]

Table S1. Segregation of homozygous *essp8* seedlings in the progeny of selfed *ESSP8/essp8* heterozygous plants

**Line Number**	**Number of plants tested**	**Percentage of homozygous seedlings**	**Percentage of non-germinating seeds**
essp8-1	132	15.9	9.1
essp8-2	112	21.4	8
essp8-3	142	16.2	4.9
essp8-4	132	19.7	5.3
essp8-5	125	20	4.8
essp8-6	123	22.8	4.1

Table S2. The percentage of defective seeds in one silique from selfed *ESSP8/essp8* heterozygous plants

**Line Number**	**Number of seeds in one silique**	**Percentage of defective seeds**
essp8-1	34	23.5
essp8-2	34	26.4
essp8-3	18	22.2
essp8-4	28	21.4
essp8-5	23	21.7
essp8-6	27	25.9

Table S3. Segregation of homozygous *gsl8* T-DNA insertion seedlings in the progeny of selfed heterozygous plants

T-DNA line	Number of plants tested	Percentage of homozygous seedlings	Percentage of non-germinating seeds
**CS801051**	205	14.2	10.5
**SALK_109342**	192	13.8	10.3
**SALK_111500**	238	18.7	6.5
**SALK_098374**	290	15.6	8.4

Table S4. List of mutant lines and primers used for genotyping

Gene	Mutant	Allele	Primer	Primer Sequence (5'-3')
GSL8	*essp8*	*essp8*	essp8-FW	TGCGTTGACCATTGTTAGCTTG
			essp8-Rev	AGGACAAAGTGGGAACGAAGAG
	SALK_111500	*gsl8-1*	GSTD-5-FW	TCTCGAATTAACGTTGTGAATCC
			GSTD-5-Rev	ACGGACATCAAACCAGTTTTG
	SALK_109342	*gsl8-2*	GSTD-7-FW	GCATCACACCAGCCTAAAATC
			GSTD-7-Rev	ATATGCTGCGATGTTTTCACC
	SAIL_21_B02	*gsl8-3*	GSTD-9-FW	GAAACCAAGTACCTCCCAAGC
			GSTD-9-Rev	CATCATCCCAGATTCGAAATG
	SALK_098374C	*gsl8-4*	GSTD-1-FW	TGTACAAAGGTGGAGTGGGAG
			GSTD-1-Rev	TCGAGTCTATGAGCTTCCAATG
	essp8 pGSL8:GSL8	GSL8	pGREEN-FW	TACGCAGTTGTCGAACTTGG
			GSL8-Rev	ACAGCATAAATCATTTTCGTTACC
			GSL8-FW	AACGGCCACGAGTTCGAGAT
			pGREEN-Rev	TATGGAACGTCAGTGGAGCA
HINKEL	SALK_056766C	*hinkel*	HNK-2-FW	ACAAATGCTAATCAGATGCCG
			HNK-2-Rev	TTAAGTCTGCACAATGTTGCC
KNOLL	SK6760	*knolle*	KNL-1-FW	AATGTGATTAGTCAAAATTTTGGG
			KNL-1-Rev	CAAACCCATCTCTGCTTTCAC
SCD1	SALK_039883	*scd1*	SCD-1-FW	GAAGTTCAGAACCACGCAGAC
			SCD-1-Rev	GGGGTGCTTCTTCATTTAAGC
KEULE	SALK_032092C	*keule*	KEUL-FW	ATGAGATTGATGGTCGTGACC
			KEUL-Rev	ATGGGACAGCGAATATTTGTG
KORRIGAN	CS6390	*korrigan*	KRG-1-FW-1	CGCGGCACAGTTTTAACGAT
			KRG-1-FW-2	GCTAATGTCGGGTGGATGGAA
			KRG-1-Rev	CTCTTCAGACGACCGTAGCC

Table S5. List of primers used for *essp8* rough-mapping

BAC	Primer	Primer Sequence (5'-3')
**F17A22**	F17A22-FW	ACGAATATTGATTGTCTAAG
	F17A22-Rev	AACCTAAGGGAAGGCTAC
**T3D7**	T3D7-FW	GGTATCGATTGAGCAAATAA
	T3D7-Rev	ACATGCGTCTGCTTGGAG
**F13M22**	F13M22-FW	AATATCCTCACGGTAAAATG
	F13M22-Rev	GGTTAAATGAAACAATTTAG
**F11F19**	F11F19-FW	GTACTGGATGTCAAACTAGA
	F11F19-Rev	ATAGCATGGTGATAAATAAG
**F19I3**	F19I3-FW	TTGTCTTAAGGGTAGTTATG
	F19I3-Rev	AGGGACTTGACGAAAGAG
**F13P17**	F13P17-FW	CTGCTGTCAAAAAAGAAGA
	F13P17-Rev	ACCTTATCCAAACAAATGTA
**F25I18**	F25I18-FW	GCGGTTCTCCTAATGAAG
	F25I18-Rev	TTTCCACGTATACTAGCA

Table S6. List of primers used for cloning and qPCR

Gene	Primer	Primer Sequence (5'-3')
SHR	SHR-FW	GGGGACAAGTTTGTACAAAAAAGCAGGCTTGAACGGTTCATTTCTGGGGCTA
	SHR-Rev	GGGGACCACTTTGTACAAGAAAGCTGGGTCCGTTGGCCGCCACGCACTA
GAPDH	qGAPDH-FW	CTTGGAAGGAGCTAGGAATTGACA
	qGAPDH-Rev	ATGTGTTTCCCTGCACCTTCTC
SCARECROW	qSCR-FW	TCTCGGAATTTACGCGGCTT
	qSCR-Rev	TGAATCGCCTGATTCGCTGT
PDLP5	PDLP5-FW	GGGGACAAGTTTGTACAAAAAAGCAGGCTTGATGATCAAGACAAAGACGA
	PDLP5-Rev	GGGGACCACTTTGTACAAGAAAGCTGGGTCTTTACACCATTTCTCATCTTG
GSL8	GSL8-FW	GGGGACAAGTTTGTACAAAAAAGCAGGCTTGATGGCTAGGGTTTATAGTA
	GSL8-Rev	GGGGACCACTTTGTACAAGAAAGCTGGGTCGGTCTCAACATTAGCTCTG
	qGSL8-FW	CTTAGGGTCCTGGGCTTGTG
	qGSL8-Rev	GAACCAGGAAAGAAGGGCGA
SCD1	SCD1-FW	GGGGACAAGTTTGTACAAAAAAGCAGGCTTGATGGGACGGATCTTCGAGTA
	SCD1-Rev	GGGGACCACTTTGTACAAGAAAGCTGGGTCGATGTTGATGGTGGCATCCC
AtBG_PPAP	AtBGPPAP-FW	GGGGACAAGTTTGTACAAAAAAGCAGGCTTGATGGCTTCTTCTTCTCTGCA
	AtBGPPAP-Rev	GGGGACCACTTTGTACAAGAAAGCTGGGTCCAACCGAAGCTTGATGATG
SUS1	SUS1-FW	GGGGACAAGTTTGTACAAAAAAGCAGGCTTGATGGCAAACGCTGAACGTAT
	SUS1-Rev	GGGGACCACTTTGTACAAGAAAGCTGGGTCATCATCTTGTGCAAGAGGAAC
UDPG	UDPG-FW	GGGGACAAGTTTGTACAAAAAAGCAGGCTTGATGGGGAAAAGAGGAAGG
	UDPG-Rev	GGGGACCACTTTGTACAAGAAAGCTGGGTCTGAATCTGCAATTTGAGACAC
GSL10	qGSL10-FW	TGGAAGCCAGTCCTAAAGCG
	qGSL10-Rev	AACCAGGAGCAGAGAGCAAC
